# Mutant p53 induces SH3BGRL expression to promote cell engulfment

**DOI:** 10.1038/s41420-025-02582-x

**Published:** 2025-07-01

**Authors:** Lobsang Dolma, Mary I. Patterson, Antonia Banyard, Callum Hall, Steven Bell, Wolfgang Breitwieser, Sudhakar Sahoo, John Weightman, Maria Pazos Gil, Garry Ashton, Caron Behan, Nicola Fullard, Lewis D. Williams, Patricia AJ. Muller

**Affiliations:** 1 Biosciences, Durham University, Durham, UK; 2 Cancer Research UK Manchester Institute, The University of Manchester, Manchester, UK

**Keywords:** Tumour-suppressor proteins, Mechanisms of disease

## Abstract

Previously, we identified that mutant p53 expression in cancer cells promotes engulfment of neighbouring cancer cells to form cell-in-cell (CIC) structures. This process gave mutant p53 cells an advantage in tumour formation in mouse xenograft experiments. *TP53* can be found mutated at nearly every amino acid in cancers and mutant p53 expression is associated with various GOF (Gain-of-function) processes, including cancer cell invasion, metastasis, stemness and drug resistance. In the current manuscript, we identified *SH3BGRL (Src homology 3 binding glutamate rich protein like*) as a mutant p53-regulated gene and investigated to what extent SH3BGRL expression and cell engulfment are responsible for mutant p53-dependent anchorage-independent growth and chemoresistance. We demonstrate that mutant p53 expression drives cell engulfment in which the mutant p53 host cell moves in the direction of the target internal cell to form CIC structures. This is therefore more reminiscent of cell engulfment rather than cell entosis, in which cells invade into host cells. Using NGS (Next Generation Sequencing), we identified novel target genes of mutant p53 and demonstrate that cell engulfment requires SH3BGRL expression. We generated mutant p53 and p53 KO cell lines that stably overexpressed SH3BGRL and determined that SH3BGRL promotes etoposide resistance in mutant p53 cells and anchorage-independent growth independent of mutant p53 expression. Through FACS sorting of pure cell engulfing (CIC) populations, we could also show that engulfing cells have an enhanced etoposide resistance. These data suggest that SH3BGRL and cell engulfment are required for certain GOFs of mutant p53.

## Introduction

p53 is a tumour suppressor regulating hundreds of target genes [1]. In response to high levels of DNA damage, p53 induces cell death, while in response to low levels, p53 allows for cell cycle arrest and DNA repair [2]. Mutations in the *TP53* gene occur in ~50–60% of human cancers [3]. *TP53* mutations can lead to either loss of p53 expression or the expression of mutant p53 proteins. Mutant proteins have been shown to lose wild-type (WT) function (Loss-of-functions) or gain additional oncogenic function (Gain-of-function, GOF). Overall, *TP53* mutation is associated with enhanced oncogenic and chemo-resistant tumours with poorer prognosis [4]. Many different mechanisms have been proposed to underlie mutant p53 GOF activity and include, but are not limited to inhibition of the p53 family members p63 and p73 [5].

Our previous work revealed a novel function for mutant p53 in promoting the formation of cell-in-cell (CIC) structures [6]. CIC structures are characterised by the presence of an internalized cell enclosed within a vacuole of a host cell bearing a distinct crescent-shaped nucleus [7, 8]. We have shown that CIC formation is associated with increased genomic instability, survival of the engulfing mutant p53 host cells and increased tumour growth [6]. In lung adenocarcinoma patients, we noted a positive correlation between mutant p53 expression and CIC formation and either mutant p53 expression or the presence of CIC structures reduced overall survival. Some other studies support a pro-tumorigenic role of CIC structures [9–11], but anti-tumorigenic roles have also been reported [12, 13].

CIC formation promotes genomic heterogeneity [6, 14], clonal selection [15] or de-selection [12], and triggers either immune evasion [16, 17] or clearance of immune cells [16, 18, 19]. In general, CIC formation can be the result of a variety of different processes involving invasion of cells into other cells (entosis) or the uptake of living or dead cells by neighbours (cannibalism or phagocytosis) [12, 20]. Mutant p53 cells were seen to extend protrusions to engulf living neighbouring cells and only in 50% of the cases did the internal cells die [6]. These findings suggest a process of direct uptake or engulfment which is distinct from an invasion process (entosis). However, the exact mechanisms underlying this process remain unknown.

In this study, we demonstrate the ability of mutant p53 to transcriptionally activate *Src homology 3 binding glutamate rich protein like* (*SH3BGRL*) to promote cell engulfment. SH3BGRL is an adaptor protein which facilitates processes including growth, signal transduction, membrane trafficking, localization of proteins to specific compartments, and cytoskeletal modifications [21–25]. It is localised in extracellular vesicles, membrane-bound organelles and the plasma membrane. Here, we show a role for SH3BGRL in promoting chemoresistance and anchorage-independent growth.

## Results

Previously, we determined that most CIC structures in cell culture were seen when mutant p53 cells were co-cultured with p53 KO cells as opposed to single cultures of mutant p53 or p53 KO cells. By examining the videos, we noticed that mutant p53 cells seem to extend protrusions to engulf p53 KO cells, suggesting the mutant p53 cells were initiating this behaviour.

**Fig. 1:**
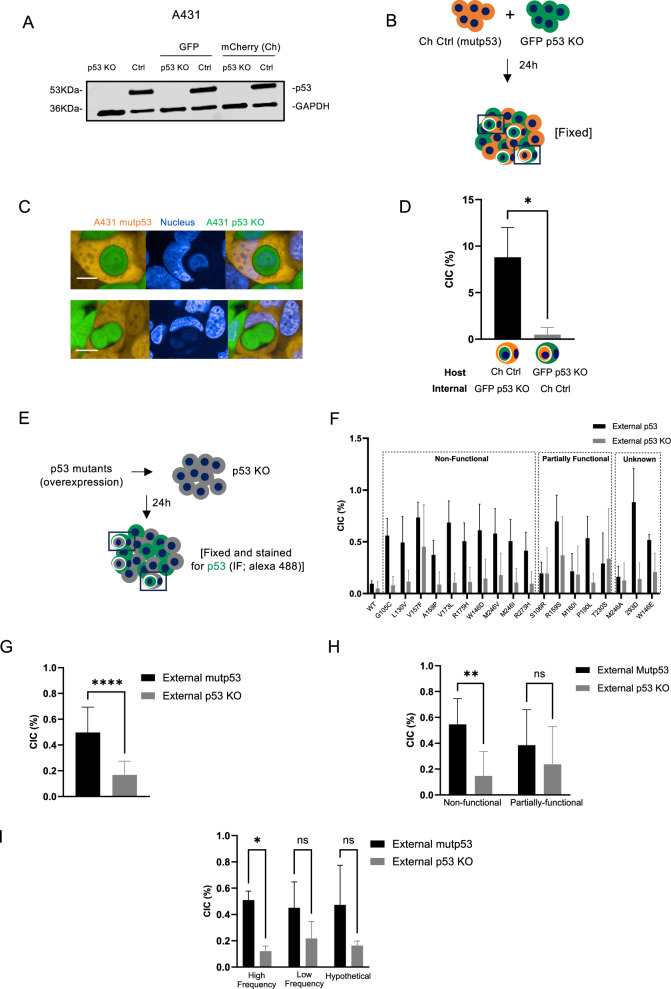
Mutant p53 often forms the outer cell in CIC structures. **A** p53 (DO-1) protein expression in A431 Ctrl (mutp53) or A431 CRISPR p53 KO cell lines. Some cell lines had a stable expression of mCherry or GFP. GAPDH was used as the loading control. **B** Schematic of experimental setup for **C**, **D**. **C** A431 mCherry Ctrl (mutp53) cells (orange) were co-cultured with GFP p53 KO cells (green) and CIC structures imaged using confocal microscopy (Opera Phenix). Blue staining is Hoechst. Scale bars indicate 20 μm. **D** Scoring of CIC structures with host cells as mCherry or GFP positive in 144 fields (*n* = 3, student’s *t*-test, *p ≤ 0.05, Error bars=SD). **E** Schematic of experimental setup of co-cultures in (**F**–**I**). **F** Non-fluorescent A431 p53 KO cells were transfected with various p53 mutants or WTp53. Cells were fixed and stained for p53 using immunofluorescence. CIC structures containing p53 positive cells were scored and displayed as cells that had p53 in the host cell (external p53) or inner cell (external p53 KO) (*n* = 3, Error bars = SD). p53 mutants were grouped as non-functional, unknown, or partially functional mutants. **G** Using all the p53 mutants shown in F, overall frequency of CIC structures with external mutp53 positive cells carrying internal p53 KO cells or external p53 KO cells carrying internal mutp53 positive cells were quantified (*n* = 3, one-way ANOVA, ****p ≤ 0.0001, Error bars = SD). **H** Non-functional mutants and partially functional mutants of F were grouped and total numbers of CIC structures in the two groups compared (*n* = 3, student’s *t*-test, **p ≤ 0.01, Error bars = SD). **I** Mutants of F were grouped based on mutational frequency and total numbers of CIC structures in the two groups compared (*n* = 3, two-way ANOVA, *p ≤ 0.05, Error bars = SD).

To study this in more detail, we used A431 cells, which endogenously express mutant p53 R273H (Ctrl mutp53) and generated p53 KO cells using CRISPR labelled with GFP or mCherry (Fig. 1A and original data) [6]. As previously seen, when combining mCherry Ctrl (mutp53) cells with GFP p53 KO cells, more mutant p53 cells were seen as outside (host) cell (Fig. 1B–D), which was independent of the fluorescence the cells expressed. Notably, numbers of CIC structures in a microscopy field are dependent on cell density and time (Supplementary Fig. 1A, B). When corrected for total cell numbers, it becomes apparent that CIC numbers normalised for cell density are relatively consistently increasing up to about 16 h, but decrease after 24 h. CIC numbers were therefore investigated between 16 and 24 h after co-culture throughout this manuscript and corrected for total cell numbers in a slide.

More CIC structures were also seen in co-cultured xenografts of A431 mCherry Ctrl (mutp53) +GFP p53 KO cells, but not in mCherry p53 KO + GFP p53 KO cells (Supplementary Fig. 1C, D). To exclude clonal selection of stable cell lines affecting CIC numbers, we transiently knocked down mutant p53 in A431 cells and could clearly observe an increased number of mutant p53-expressing external cells in the CIC structures (Supplementary Fig. 1E–I and original data). These data demonstrate that mutant p53 drives cell engulfment in A431 cells.

To determine if the type of p53 mutation influences CIC formation, we expressed a random panel of different p53 mutants annotated on the *TP53* database (Supplementary Table 1) [26]. As frequent mutations are more likely selected for and therefore more associated with aggressive cancers, we selected mutants that are frequently or infrequently observed in cancers and a few hypothetical mutants, based on the p53 database. The majority of the p53 mutants, but not WT p53 were observed as the outside cell of the CIC structures (Fig. 1E, F). Note that the overall numbers of CIC are lower than in stable clones (Fig. 1D), which we note to be an effect of transfections. Looking at all the mutants together, mutant p53 expression is significantly correlated to outer cell status (Fig. 1G). Interestingly, highly frequent mutants and those mutants that are classed as non-functional mutants according to the *TP53* database were more likely associated with host cell status than those classed as infrequent or partially-functional (retaining some WT function) (Fig. 1F, H and I).

These data suggest mutant p53 is initiating CIC formation, but do not discriminate between mutant p53 cells engulfing p53 KO cells or mutant p53 cells becoming more deformable allowing p53 KO cells to invade into them via an entotic process. Invasion or entosis has previously been shown dependent on higher RhoA expression in the invading inner cells [15]. To examine to what extent CIC formation of A431 cells was entosis-dependent, we assessed endogenous RhoA expression and expressed GFP-RhoA in A431 Ctrl (mutp53) or p53 KO cells (Supplementary Fig. 2A–C). Endogenous RhoA expression was equal in mutant p53 compared to p53 KO cells (Supplementary Fig. 2A and original data). In both cell lines, overexpression of RhoA resulted in an increased number of CIC structures that had RhoA overexpressed in internal cells (Supplementary Fig. 2B, C). These data show that A431 cells can form CIC structures in an entotic manner and that RhoA promotes entosis in A431 cells. However, no difference was observed in the number of RhoA-positive inner cells in mutant p53 compared to p53 KO cells. It is therefore less likely that mutant p53 status allows for a more deformable membrane, suggesting CIC formation by mutant p53 does not occur through entosis.

**Fig. 2:**
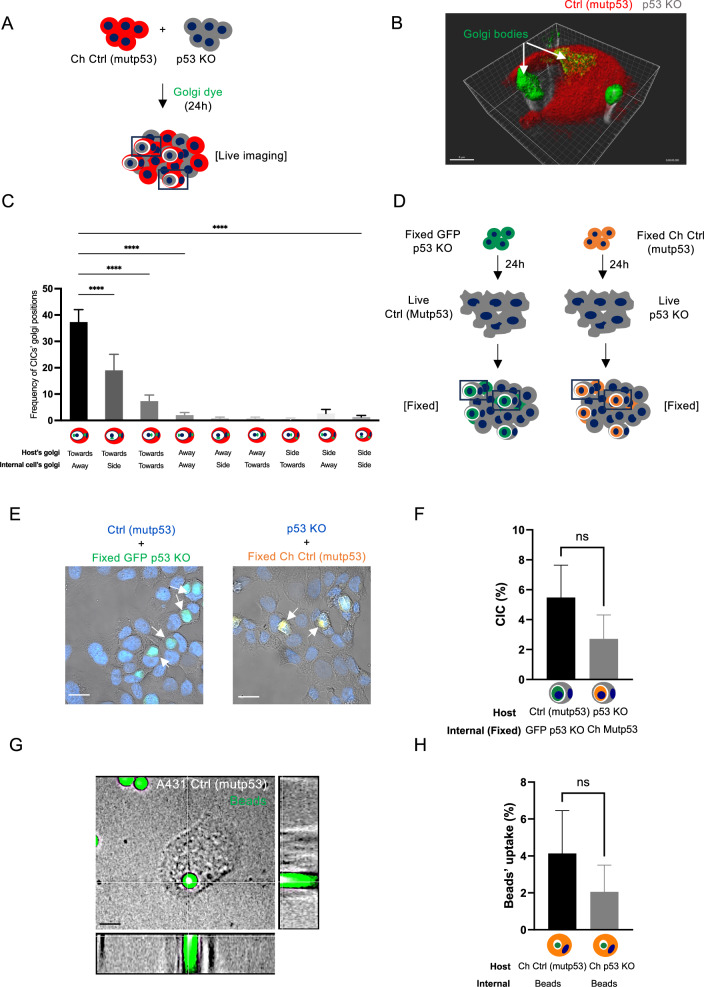
Mutant p53 status drives cell engulfment. **A** Schematic of the experimental set up for B. **B** A431 mCherry Ctrl (mutp53) cells (red) were co-cultured with non-fluorescent p53 KO cells (grey), exposed to live Golgi-tracker dye (green) and live time-series imaging was done. A z-stack (46.08 μm in 193 planes) of the live cells was taken on the Andor spinning disk microscope (scale bar = 8 μm) and 3D image generated using Imaris image analysis software (version 9.2) (Oxford instruments). **C** A431 mCherry Ctrl (mutp53) cells were co-cultured with non-fluorescent p53 KO cells and fixed for immunofluorescence. The Golgi apparatus was stained with GM130, and CIC structures quantified based on the orientation of the host and internal cell Golgi as indicated at the bottom of the graph (*n* = 3, one-way ANOVA, ****p ≤ 0.0001, Error bars = SD). **D** Schematic of the experimental setup for **(E**). Non fluorescent A431 Ctrl (mutp53) cells and p53 KO cells were cultured in the presence of fixed GFP p53 KO or mCherry Ctrl (mutp53) cells, respectively. **E** Example images of CIC structures indicated by arrows (Zeiss LSM800 microscope, scale bar = 30 μm). **F** Quantification of A431 Ctrl (mutp53) and p53 KO cell ability to engulf fixed GFP p53 KO or mCherry Ctrl (mutp53) cells, respectively (*n* = 3, student’s *t*-test, Error bars=SD). A431 mCherry Ctrl (mutp53) or mCherry p53 KO cells were cultured in the presence of green, fluorescent beads. **G** Bead (5μm) uptake was imaged using confocal microscopy (Spinning disk). Blue staining is Hoechst and scale bars represent 30 μm. **H** Bead uptake was also measured using flow cytometry (*n* = 3, student’s *t*-test, Error bars = SD).

To determine if mutant p53 cells purposely move towards p53 KO cells, we examined the localisation of the Golgi apparatus. Motile cells orient their Golgi in the direction in which they move [27]. Using live Golgi tracker dye in A431 mCherry Ctrl (mutp53) cells co-cultured with non-fluorescent A431 p53 KO cells, we could see the Golgi apparatus of the host Ctrl (mutp53) cell oriented towards the internal p53 KO cell. At the same time, the Golgi apparatus of the internal p53 KO cell was facing away during CIC formation (Fig. 2A, B). To quantify this, we stained A431 cell co-cultures (mCherry Ctrl (mutp53) + p53 KO) for GM130 (Golgi staining) and determined the localisation of the Golgi in the CIC structures (Fig. 2C). Most host cells oriented their Golgi towards the cell that is being engulfed. In contrast, engulfed cells can have their Golgi oriented in all directions, although it appears many are facing away from the host cell (Fig. 2C).

If mutant p53 cells drive the process of cell engulfment, they may engulf fixed cells (cells that are fixed upon trypsinisation to not express apoptotic markers) (Fig. 2D). Fixed A431 GFP p53 KO or fixed mCherry (Ch) Ctrl (mutp53) cell uptake in non-fluorescent Ctrl (mutp53) or p53 KO cells was determined using Hoechst/GFP or Hoechst/mCherry expression (Fig. 2E, F). Engulfment of fixed cells was slightly more pronounced in Ctrl (mutp53) cells, although not significantly. Directly comparing these results to Fig. 1D, we can see that the overall capacity of engulfment by mutant p53 is decreased when exposing them to ‘fixed’ cells and that the engulfment of ‘fixed’ cells by p53 KO cells is slightly enhanced (Supplementary Fig. 2D). Uptake of fluorescent beads could be observed in A431 cells but was also not significantly different between A431 Ctrl (mutp53) or p53 KO cells (Supplementary Video 1, Fig. 2G, H). These data suggest that A431 cells are inherently able to engulf, but that the capacity to engulf can be influenced by signalling or intrinsic differences between the two cells.

**Fig. 3:**
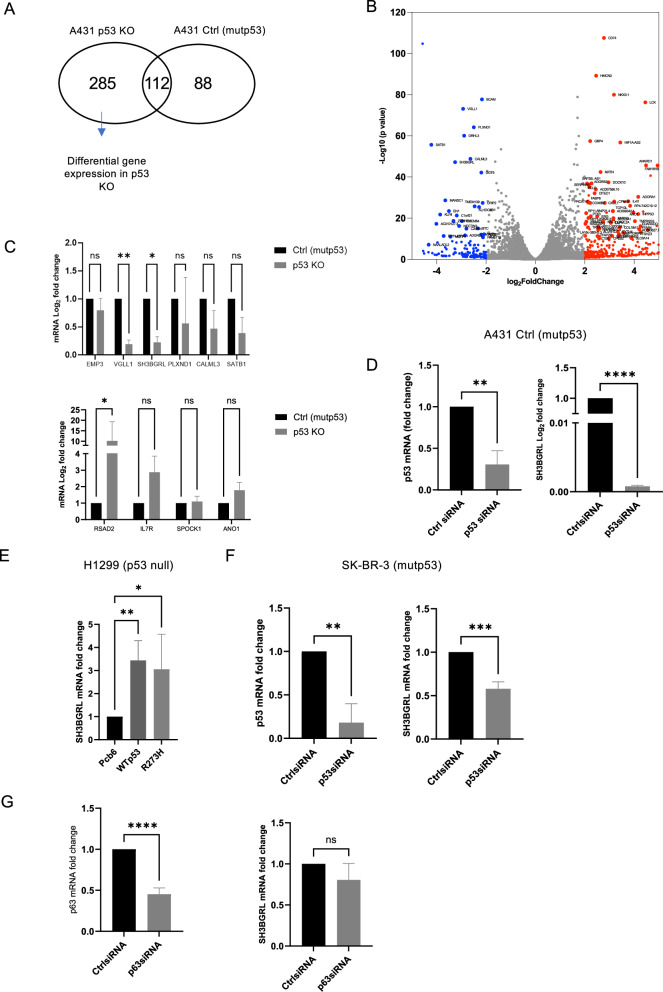
*SH3BGRL* expression is driven by mutant p53. **A** Differential gene expression in A431 p53 KO cells was determined using NGS using parental (mutp53) and A431 Ctrl (mutp53) cells as controls. Indicated are the number of genes identified as differentially expressed (1.5 log_2_ fold) with the parental A431 cells. **B** Genes that are up or downregulated by more than 1.5 log_2_ fold change in A431 p53 KO cells compared to both A431 parental (mutp53) and Ctrl (mutp53) cells are depicted in red (upregulated) or blue (downregulated) in the Volcano plot. **C** Gene expression of potential CIC-relevant up (top) or downregulated (bottom) genes was validated through qRT PCR in independent lysates of A431 Ctrl (mutp53) and p53 KO cells (*n* = 3, two-way ANOVA, **p ≤ 0.01, *p ≤ 0.05, Error bars=SD). **D** A431 parental (mutp53) cells were transfected with p53 siRNA and *TP53* (left) and *SH3BGRL* (right) gene expression measured using qRT PCR (*n* = 3, student’s *t*-test, **p ≤ 0.01, ****p ≤ 0.0001, Error bars=SD). **E** H1299 (p53 null) cells were transfected with a control (Pcb6) or mutant p53 (R273H) and examined for *SH3BGRL* mRNA expression using qRT PCR (*n* = 3, one-way ANOVA,*p ≤ 0.05, **p ≤ 0.01, Error bars = SD). **F** SK-BR-3 cells which endogenously express mutant p53 (R175H) were transfected with p53 siRNA or Ctrl siRNA and *TP53* (left) and *SH3BGRL* (right) gene expression measured using qRT PCR (*n* = 3, student’s *t*-test, **p ≤ 0.01, ***p ≤ 0.001, Error bars=SD). **G** H1299 parental cells were transfected with a control or p63 siRNA and examined for *SH3BGRL* (right) or *TAp63* (left) mRNA expression using qRT PCR (*n* = 3, student’s *t*-test, ****p ≤ 0.0001, Error bars = SD).

Many of the GOFs of mutant p53 rely on mutant p53-mediated regulation of transcription factors such as p63, p73 and many others [28]. We therefore used Next Generation Sequencing (NGS) of A431 parental, A431 Ctrl (mutp53) and A431 p53 KO cells to determine which genes are differentially expressed in p53 KO cells (Fig. 3A). We identified genes that were significantly upregulated and genes that are significantly downregulated by greater than 1.5 log_2_ fold change in p53 KO cells (Supplementary Table 2, Fig. 3B). Pathway enrichment analysis showed pathways such as interleukin signalling, extracellular matrix degradation and signalling by WNT as differentially enriched in p53 KO cells (Supplementary Fig. 2E) [29]. Of the top 50 genes, we selected the first 5 most up and most downregulated genes whose expression changes have been associated with aggressive breast or lung cancer. We selected these cancers as the presence of CIC structures correlates to worse survival [6, 30]. After validating by qRT PCR, only *VGLL1*, *SH3BGRL* and *RSAD2* were significantly down or upregulated in p53 KO cells compared to control A431 cells in a similar manner as in the NGS screen (Fig. 3C). We next determined if we could further validate these genes using siRNA mediated knockdown of p53 in the parental A431 cells. *SH3BGRL* expression significantly decreased more than tenfold (Fig. 3D, right) in response to p53 knockdown as validated by qRT-PCR (Fig. 3D, left). We expected a decrease of *VGLL1* expression and an increase in *RSAD2* expression upon loss of p53, but if anything, we saw the opposite response with a remarkable tenfold decrease in *RSAD2* expression, potentially suggesting clonal variations (Supplementary Fig. 2F). We therefore only followed up on *SH3BGRL* expression in other cell lines. In H1299, stable expression of mutant p53 R273H resulted in increased *SH3BGRL* expression (Fig. 3E). Remarkably, we also saw an increase when overexpressing WT p53, which could indicate that mutant p53 273H-mediated expression of *SH3BGRL* is a remnant WT function. Knockdown of p53 in SK-BR-3 cells, expressing R175H mutant p53, decreased *SH3BGRL* expression (Fig. 3F). Previous GOF effects of mutant p53 expression in H1299 were shown to be dependent on mutant p53-mediated repression of *TAp63* [31]. We therefore knocked down p63 in H1299 cells and examined if *TAp63* regulates *SH3BGRL* expression. *SH3BGRL* expression did not significantly change (Fig. 3G), suggesting that mutant p53 does not regulate *SH3BGRL* via *TAp63* inhibition.

p53 mutations are strongly correlated with survival in all cancers (Supplementary Fig. 3A). *SH3BGRL* expression in all cancers is higher than in a non-diseased population (Supplementary Fig. 3B). We therefore speculated *SH3BGRL* expression would be correlated to survival, however, in contrast to what we predicted, a better survival of patients with high *SH3BGRL* expression in all cancers was seen (Supplementary Fig. 3C), which was independent of p53 mutation status (Supplementary Fig. 3D). CIC structures are widely observed in epithelial cancers and we therefore wondered if a correlation between *SH3BGRL* expression, survival and p53 mutations would be more obvious in carcinomas. *SH3BGRL* expression is higher in carcinomas compared to expression in non-diseased epithelial tissues (Supplementary Fig. 3E). However, *SH3BGRL* expression was significantly associated with better survival in WTp53 expressing patients, but not in patients that had TP53 mutations (Supplementary Fig. 3F). Previous results in breast cancer suggest *SH3BGRL* expression is correlated to worse prognosis [24, 32]. We therefore examined *SH3BGRL* expression in breast invasive carcinoma and detected a significant higher expression of *SH3BGRL* in these cancers compared to non-diseased breast tissue (Supplementary Fig. 3G). We were not able to determine a correlation with p53 status, *SH3BGRL* and survival in these datasets, which is likely due to limits in numbers of patients in this database (Supplementary Fig. 3H). These data suggest that SH3BGRL expression is likely regulated by more than p53 status in these cancers.

**Fig. 4:**
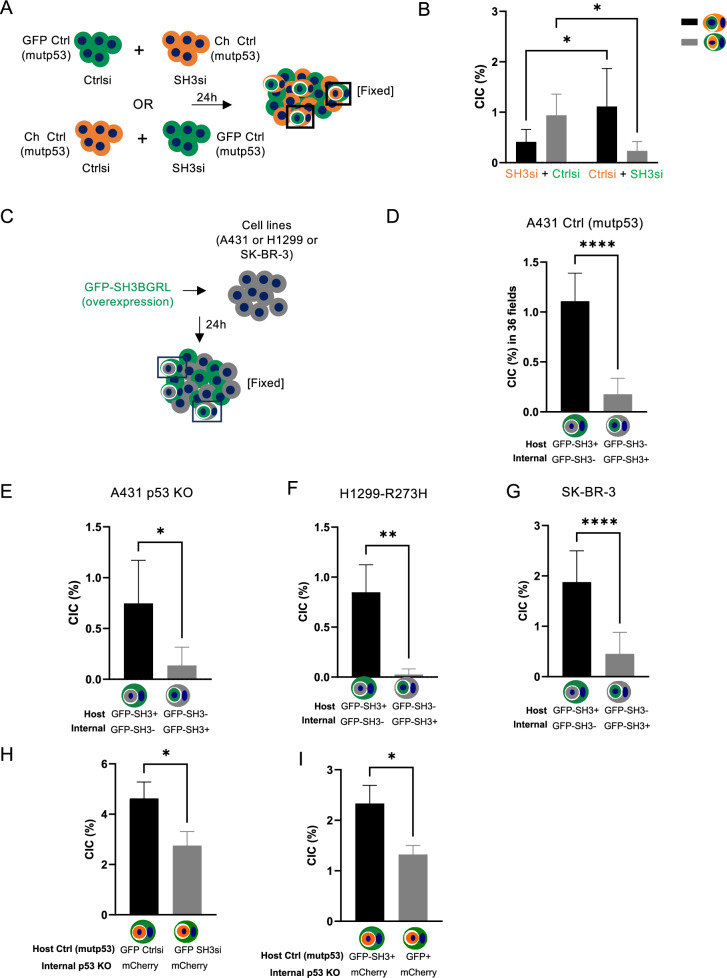
SH3BGRL is found expressed in outer cells in CIC structures. **A** Schematic of co-cultures used in B. **B** A431 mCherry Ctrl (mutp53) (orange) or GFP Ctrl (mutp53) (green) cells were transfected with SH3BGRL siRNA or Ctrl siRNA as depicted in A and CIC structures examined through confocal microscopy (Zeiss LSM800). CIC structures with host GFP-labelled cells carrying mCherry-labelled cells or host mCherry-labelled cells taking up GFP-labelled cells were scored and depicted (*n* = 3, two-way ANOVA, *p ≤ 0.05, Error bars = SD). **C** Schematic of non-fluorescent cell co-cultures transfected with GFP-SH3BGRL used in (**D**–**G**). A431 Ctrl (mutp53) cells (**D**), A431 p53 KO cells (**E**), H1299 cells with stable expression of mutant p53 (R273H) (**F**) and SK-BR-3 cells (**G**) were transfected with GFP-tagged SH3BGRL after which CIC structures consisting of host or internal GFP positive cells were scored (*n* = 3, student’s *t*-test, *p ≤ 0.05, **p ≤ 0.01, ****p ≤ 0.0001, Error bars = SD). **H** A431 GFP Ctrl (mutp53) cells were transfected with Ctrl siRNA or SH3BGRL siRNA and subsequently co-cultured with mCherry p53 KO cells. CIC structures with host GFP Ctrl (mutp53) cells carrying internal mCherry p53 KO cells were scored after 24 h (*n* = 3, student’s *t*-test, *p ≤ 0.05, Error bars=SEM). **I** A431 non-fluorescent Ctrl (mutp53) cells were overexpressed with GFP or GFP-SH3BGRL and co-cultured with mCherry p53 KO cells for CIC quantification. CICs with host GFP or GFP-SH3BGRL Ctrl (mutp53) cells and internal mCherry p53 KO cells were quantified (*n* = 3, student’s *t*-test, *p ≤ 0.05, Error bars=SEM).

We next investigated to what extent SH3BGRL was involved in mutant p53-mediated cell engulfment. We knocked down SH3BGRL in A431 mCherry Ctrl (mutp53) or GFP Ctrl (mutp53) cells (Supplementary Fig. 4A) and determined the number of CIC structures in which SH3BGRL was expressed in the host or inner cell (Fig. 4A, B). In each co-culture, cells that had lost *SH3BGRL* expression were more likely to end up as inner cell (Fig. 4B). We next expressed GFP-SH3BGRL in non-fluorescent A431 Ctrl (mutp53) and A431 p53 KO cells (Fig. 4C–E) and identified that most CIC structures had GFP positive cells as the host cell, suggesting that *SH3BGRL* expression favours engulfment (Fig. 4D, E). This happened regardless of p53 status although the total number of CIC structures in p53 KO cells remained lower than in Ctrl (mutp53) cells (Fig. 4D, E). SH3BGRL-expressing cells were also more frequently observed as outer cells in CIC structures of H1299 cells (mutant p53 R273H) and SK-BR-3 (mutant p53 R175H) cells (Fig. 4F, G). These experiments show that SH3BGRL expression can identify host cell status, but do not necessarily show that SH3BGRL promotes cell engulfment. We therefore knocked down (Fig. 4H) or overexpressed (Fig. 4I) SH3BGRL in GFP mutant p53 cells and combined these with mCherry p53 KO cells. These results clearly illustrate that SH3BGRL not only identifies host cells status but also promotes cell engulfment in a mutant p53 setting (Fig. 4H, I).

**Fig. 5:**
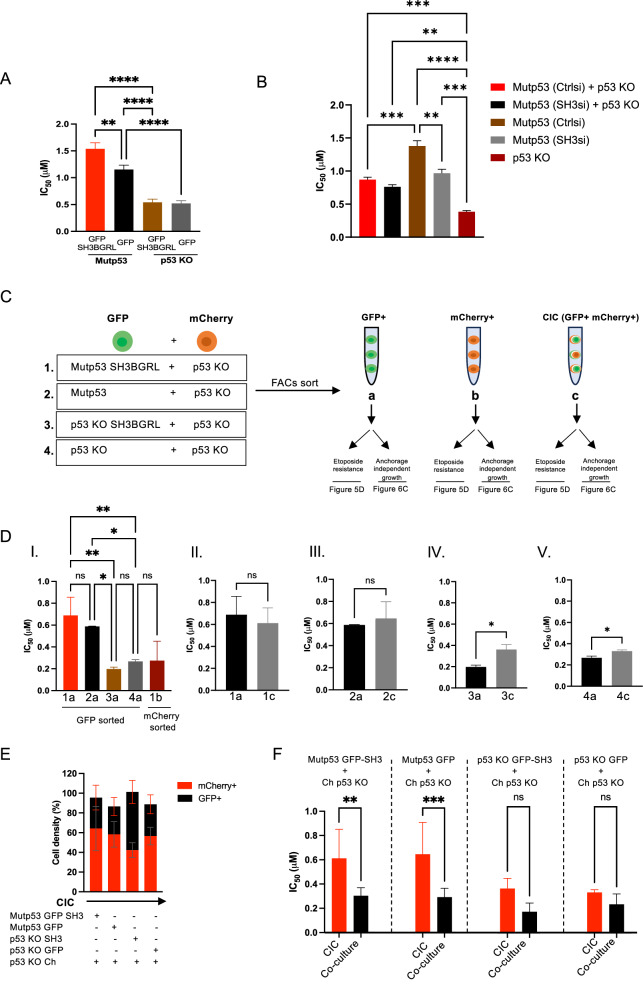
SH3BGRL and CIC structures independently of each other promote etoposide resistance in mutant p53 cells. **A** A431 Ctrl (mutp53) or p53 KO cells stably expressing GFP-SH3BGRL or GFP were grown in resazurin survival assays and treated with increasing doses of etoposide. Cell survival (%) after 72 h was determined through non-linear regression model (inhibitor vs normalised response) and IC_50_ calculated (*n* = 3, two-way ANOVA, **p ≤ 0.01, ****p ≤ 0.0001, Error bars = SEM). **B** A431 Ctrl (mutp53)cells with or without SH3BGRL knockdown were either grown on their own or co-cultured with p53 KO cells in survival assays. Equal numbers of cells were used in each condition, meaning that a co-culture has half the number of mutant cells than the single mutant p53 condition. Cell survival (%) after 72 h was determined through non-linear regression model (inhibitor vs normalised response) and IC_50_ calculated (*n* = 3, two-way ANOVA, **p ≤ 0.01,***p ≤ 0.001, ****p ≤ 0.0001, Error bars=SEM). **C** Schematics of the co-cultures incorporated for FACs sorting of GFP+, mCherry+ and double positive CIC populations used in D–F. **D** IC_50_ of etoposide survival assays as measured in the sorted cell populations of C (*n* = 3, two-way ANOVA, *p ≤ 0.05, **p ≤0.01, Error bars = SEM). 1a, 2a, 3a and 4a represent FACS sorted Ctrl (mutp53) GFP-SH3, Ctrl (mutp53) GFP, p53 KO GFP-SH3 and p53 KO GFP, respectively. 1b indicates FACS sorted mcherry p53 KO cells. 1c,2c, 3c and 4c represent FACS sorted CIC populations from mutp53 (GFP-SH3 or GFP) + mCherry p53 KO or p53 KO (GFP-SH3 or GFP) +mCherry p53 KO co-cultures as indicated in (**C**). **E** Percentage of red (mCherry) and green (GFP) cells in each CIC population shown in (**D**). **F** IC_50_ of etoposide survival assays on FACS sorted CIC populations or co-cultures that were set up only prior to etoposide survival assays based on the ratio of red and green cells in (**E**) (*n* = 3, two-way ANOVA, **p ≤ 0.01, ***p ≤ 0.001, Error bars = SEM).

Mutant p53 has previously been shown to promote invasion, cell migration and chemoresistance [29, 31]. We first explored chemoresistance and examined cell viability of A431 Ctrl (mutp53) and p53 KO cells with or without GFP-SH3BGRL overexpression when exposed to increasing etoposide concentrations. As expected, p53 KO cells were more sensitive to etoposide than mutant p53 cells (Fig. 5A and Supplementary Fig. 5A). GFP-SH3BGRL expression did not cause chemoresistance in p53 KO cells, but it exacerbated chemoresistance in Ctrl (mutp53) cells (Fig. 5A and Supplementary Fig. 5A). Loss of SH3BGRL in mutant p53 cells reduced etoposide resistance (Fig. 5B), suggesting mutant p53 requires SH3BGRL to promote etoposide resistance, but only SH3BGRL overexpression promotes etoposide resistance in a mutant p53 setting.

We next investigated whether mutant p53/ SH3BGRL-dependent resistance could be the result of CIC formation. We therefore co-cultured mutant p53 cells with or without SH3BGRL expression and p53 KO cells assuming that co-cultures are correlated to more engulfment. The measured etoposide resistance is approximately the average between what is seen for mutant p53 cells or p53 KO cells alone (Fig. 5B), suggesting that co-culturing these cells does not significantly contribute to etoposide resistance. Notably, loss of SH3BGRL in the mutant p53 cells in these co-cultures did not significantly decrease etoposide resistance (Fig. 5B), but this significance might have been lost due to relying on smaller differences in etoposide resistance due to dilution of co-cultures.

Importantly, in these experiments, we have to consider that the percentage of CIC in these specific co-cultures is low, estimated at less than 1%, as we cannot start with many cells to be able to do the survival for 72 h. In addition, co-cultures can be affected by cell signalling and cell-cell contacts that are not necessarily related to CIC formation. We therefore used FACS sorting to try and select specifically for CIC structures. We isolated CIC structures of GFP and mCherry labelled A431 Ctrl (mutp53) and p53 KO co-cultures as schematically indicated in Fig. 5C. FACS was optimized using Imagestream MKII in which flow cytometry is combined with live imaging of fluorescent cells, which allowed for setting up the best parameters to select engulfing cells. Single cells were first selected (Supplementary Fig. 5B) to then gate on focused cells only (Supplementary Fig. 5C). The double positive cells (GFP and mCherry) were quantified (Supplementary Fig. 5D) and validated as actual CIC events by image analysis (Supplementary Fig. 5E) and first gating for ‘single and rounded’ engulfing cells using brightfield’s area major axis (*y* axis) and area (*x*-axis) (Supplementary Fig. 5F). From those, 90.85% were validated as true CIC structures, based on image analysis (Supplementary Fig. 5G, H). We validated this method using A431 mCherry Ctrl (mutp53) cells co-cultured with GFP p53 KO cells and detected that 96% of the CIC structures constituted mCherry Ctrl (mutp53) cells with inner GFP p53 KO cells whilst only 4% were GFP p53 KO cells with inner mCherry Ctrl (mutp53) cells (Supplementary Fig. 5G, H).

We compared FACS-sorted A431 GFP Ctrl (mutp53) cells, FACS-sorted GFP and mCherry p53 KO cells and FACS-sorted CIC structures (GFP and mCherry positive) from the various co-cultures (Fig. 5C) for etoposide resistance. After cell sorting, cells were left to grow for four days, passaged and assessed for etoposide resistance using cell viability assays. Importantly, CIC-sorted cells retained the capacity to engulf, but did not show an increase in numbers of CIC after this time suggesting that CIC is not performed by a subset of cells that we selected for by sorting (Supplementary Fig. 5I).

Etoposide resistance was still prominent in the GFP Ctrl (mutp53) cells compared to GFP or mCherry p53 KO cells (Fig. 5D I. 2a vs 4a), although overall resistance of all cells was lower compared to viability studies in Fig. 5A. Remarkably, sorted CIC structures showed a similar resistance to sorted Ctrl (mutp53) cells (Fig. 5D II-III.). At first glance, it appears as if CIC-sorted populations do not display etoposide resistance, however, it is important to note that the sorted co-cultures consist of ~60% mCherry p53 KO cells (Fig. 5E, column 1 and 2). Considering the twofold reduced etoposide resistance seen for co-cultures in Fig. 5B, we expected to see a 60% reduction. In fact, direct comparison of sorted CIC structures as in 5D (1c, 2c, 3c and 4c) to cells freshly mixed in a 60:40 (p53 KO: Ctrl (mutp53)) ratio clearly illustrates that sorted mutant p53 CIC populations are more resistant to etoposide (Fig. 5F). Importantly, CIC mediated etoposide resistance did not seem to be SH3BGRL dependent (Fig. 5D–F).

On similar lines, sorted p53 KO CIC populations appear at first glance to marginally increase chemoresistance (Fig. 5D IV–V). However, this increase was lost when compared to direct co-cultures mixed in the same ratio. In fact, CIC-sorted p53 KO cells were more resistant to etoposide, but this was not significant. In addition, SH3BGRL expression could not further enhance chemoresistance in p53 KO cells. These results suggest that sorted CIC might enhance etoposide resistance, but this is independent of SH3BGRL expression.

**Fig. 6:**
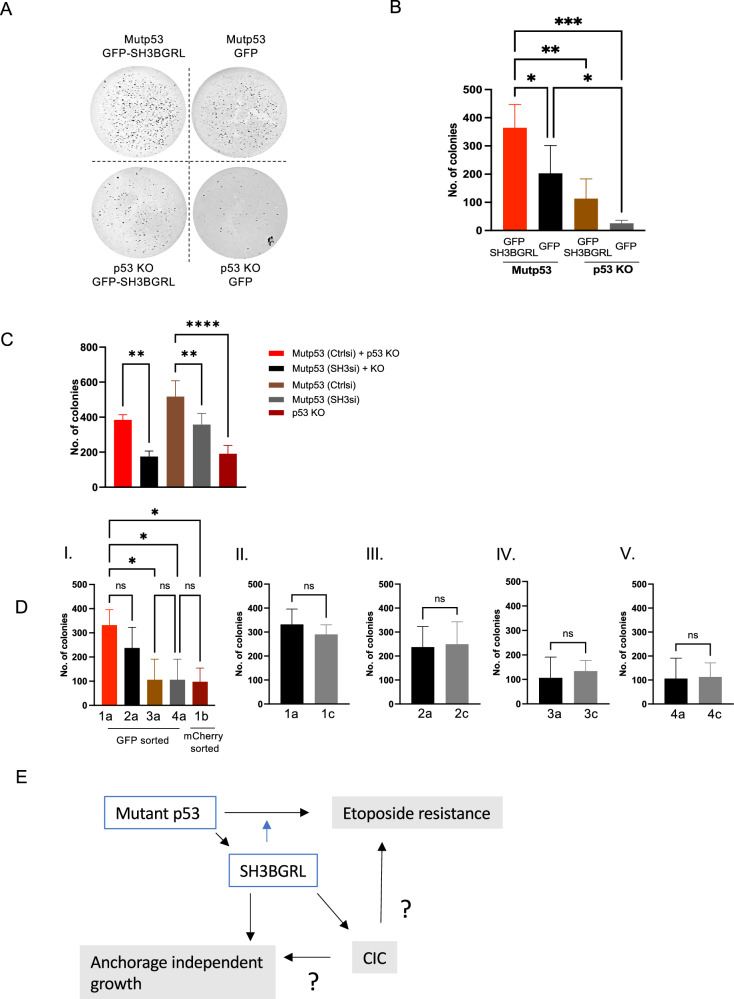
SH3BGRL and CIC structures independently of each other promote anchorage-independent growth. A431 Ctrl (mutp53) or p53 KO cells stably expressing GFP-SH3BGRL or GFP were grown in anchorage independent growth assays, imaged (**A**) and quantified for numbers of colonies (**B**) (*n* = 3, two-way ANOVA, *p ≤ 0.05, **p ≤ 0.01, ***p ≤ 0.001, Error bars = SD). **C** A431 Ctrl (mutp53) cells with or without SH3BGRL knockdown were grown on their own or co-cultured with p53 KO cells in anchorage-independent growth assays and colony formation was measured after 14 days (*n* = 3, two-way ANOVA,**p ≤ 0.01, ***p ≤ 0.001, Error bars = SD). **D** FACS-sorted cell populations in Fig. 5C were also studied for their ability to grow in anchorage-independent growth assays. The number of colonies are quantified (*n* = 3, two-way ANOVA, *p ≤ 0.05, Error bars = SD). **E** Graphical representation of mutant p53’s function in regulating CIC formation and SH3BGRL expression. Mutant p53 promotes SH3BGRL expression that can assist mutant p53 mediated etoposide resistance and promote anchorage-independent growth. CIC formation is likely to contribute to etoposide resistance and possibly to anchorage-dependent growth, but likely in a SH3BGRL-independent manner.

We next assessed another mutant p53 GOF, anchorage-independent growth. Using the A431 Ctrl (mutp53) or p53 KO cells with or without GFP-SH3BGRL overexpression, we validated that mutant p53 promotes anchorage-independent growth (Fig. 6A, B). Interestingly, SH3BGRL overexpression enhanced the capacity of both A431 Ctrl (mutp53) and p53 KO cells to grow in soft agar (Fig. 6B) and loss of SH3BGRL expression in mutant p53 cells reduced colony formation (Fig. 6C).

To assess the role of CIC formation in anchorage-independent growth, we first combined co-cultures (Fig. 6C). We noticed that co-cultures of mutant p53 and p53 KO cells formed less colonies than mutant p53 cells alone, but this was not significant. Loss of SH3BGRL in the mutant p53 cells of these co-cultures further reduced the number of colonies. As for Fig. 6C, cells in agar are at a low density and we therefore weren’t sure if co-cultures represented cell engulfment well enough to conclude that CIC formation does not contribute to colony formation in anchorage-independent growth assays.

We therefore also used the sorted cells of Fig. 5D, E. Notably FACS sorting affects the capacity of cells to form colonies as in all conditions fewer and smaller colonies were detected, which made it hard to compare these results to non-sorted cells. A431 Ctrl (mutp53) cells still grew better than p53 KO cells in soft agar and SH3BGRL expression further enhanced this growth (Fig. 6D 1a and 2a compared to 3a, 4a and 1b). Notably, increased anchorage-independent growth of p53 KO cells upon SH3BGRL overexpression was not observed in the FACS sorted population of p53 KO cells, which could reflect that co-culture has changed the ability of these cells to grow in anchorage-independent growth. Sorted CIC structures showed a capacity as high as mutant p53 cells in inducing growth in soft agar (Fig. 6D II-III), which given the higher proportion of p53 KO cells in this culture (Fig. 5E) may be the result of cell engulfment. SH3BGRL overexpression in any of the sorted CIC structures did not further enhance anchorage-independent growth (Fig. 6D 1c compared to 2c and 3c compared to 4c). These data suggest that SH3BGRL promotes anchorage-independent growth, likely in a more direct manner and likely not via CIC formation (Fig. 6E).

In conclusion, mutant p53 regulates SH3BGRL expression, which assists mutant p53 in promoting etoposide resistance, and promotes anchorage-independent growth. CIC formation appears to promote etoposide resistance and it may be involved in anchorage-independent growth, but this is likely independent of SH3BGRL regulation (Fig. 6C).

## Discussion

In this manuscript, we have carefully assessed the role of mutant p53 cells in driving CIC formation. Our process resembles a more phagocytic type of engulfment that causes mutant p53 cells to move towards p53 KO cells. In addition, A431 cells can engulf fixed cells or beads, suggesting this epithelial cell line has gained some phagocytic activity to engulf neighbouring cells. Interestingly, engulfment of beads or fixed cells seemed independent of mutant p53 status, suggesting that cell-intrinsic differences between the two cells, additional signalling or cell-cell contacts contribute to engulfment.

Previously, we described that mutant p53 cells were more likely to survive cell division when carrying an engulfed cell, but that the capacity to engulf was similar between p53 KO cells and mutant p53 cells [6]. Perhaps fixed cells are easier to degrade and perhaps the beads are small enough not to interfere in cell division. Many factors can affect the total number of CICs that you can detect in a cell culture. Unsurprisingly, these include cell density and time after seeding as we show in Supplementary Fig. 1A, B. In addition, we noticed that transfections cause a lower number of CICs to be successfully formed, possibly as a result of affecting the stiffness or deformability of cell membranes. Future work will focus on finding the optimal parameters as well as the differences between uptake of cells, various particles and cargo and to understand the mechanisms underlying cell engulfment.

A genome-wide NGS experiment was performed to determine differential gene expression in mutant p53 cancer cells compared to p53 KO cancer cells. These data revealed that *SH3BGRL* is transcriptionally regulated by mutant p53. Similar to mutant p53 expressing cells, SH3BGRL-positive cells were more frequently observed as the outer cells in CIC structures and in co-cultures we demonstrate that loss of SH3BGRL in mutant p53 cells reduces the number of CIC structures. *SH3BGRL* expression has been associated with cancer formation, promoting tumour initiation via c-src in murine cell lines [33], but also with tumour suppression, preventing tumour formation of various human cancer cells [33, 34]. It is often found overexpressed in cancers compared to normal tissues [33], although some cancers show a lower expression, e.g. liver cancer [34]. Similarly, high *SH3BGRL* expression can both be associated with a better survival [35, 36] or with a reduced survival [24, 32, 37, 38] of cancer patients. These data could highlight that SH3BGRL plays a different role in different cancers. The findings that in AML, SH3BGRL was both identified as a tumour promoter and a tumour suppressor [36, 39], suggest that even factors within the same cancer can make SH3BGRL behave differently. One such factor could be mutations in SH3BGRL itself as was described by Wang et al. [33]. Mouse and human SH3BGRL differ by a V108 mutation that can turn this protein from an oncogene into a tumour suppressor. Other factors could be environment factors or the presence of oncogenes or tumour suppressors, including p53 status.

In general, p53 mutations are associated with worse survival in all cancer patients (Supplementary Fig. 3A). Our results showed that high expression of *SH3BGRL* was associated with a better survival in all cancers (Supplementary Fig. 3C). When stratified for p53 mutations, this was seen in both WTp53 and in mutant p53 cancers, suggesting that mutant p53 might not always regulate *SH3BGRL* expression. Some p53 mutations retain WT function and we discovered that WT p53 can also regulate *SH3BGRL* expression, suggesting that *SH3BGRL* regulation is a remnant WT activity. However, WT p53 did not promote CIC formation, suggesting WT p53 independently of SH3BGRL inhibits engulfment. Others have seen WT p53 initiating the process of entosis [40]. Future research in our lab will focus on delineating the differences between entosis and engulfment and examining the role of WT p53 and p53 mutants in this process. Within histological sections we currently cannot discriminate if a CIC was formed through entosis or engulfment and with this research we hope to identify biomarkers to discriminate between these processes.

Another consideration regarding p53 status and correlations to expression of genes such as *SH3BGRL* in cancer is that p53 activity can be influenced by external factors. Hypoxia or increased metal levels are known to unfold the p53 molecule and inactivate it [41]. Our lab is currently investigating the transcriptional changes upon p53 unfolding, which suggest that some genes might actually be regulated by unfolded p53. Finding biomarkers associated with unfolding of p53 is something that our lab is currently investigating and might help to determine a correlation between p53 function and genes such as *SH3BGRL* in cancers.

We next investigated to what extent mutant p53-induced SH3BGRL expression and CIC formation are responsible for mutant p53 GOF phenotypes for etoposide resistance and anchorage-independent growth [29, 31]. SH3BGRL promoted etoposide resistance only in mutant p53 cells and not in p53 KO cells. Other researchers have implicated CIC structures and SH3BGRL function in chemoresistance. SH3BGRL promotes chemoresistance by promoting autophagy [37]. Interestingly, mutant p53 can activate autophagy in various ways [42] so perhaps mutant p53 enables SH3BGRL to promote etoposide resistance through cytoprotective autophagy. Autophagy has been associated with entotic clearance of cells, in which the autophagy machine is recruited to mediate the degradation of the internal cell upon cell death [43]. However, as we do not see enhanced cell death of the internal cell upon mutant p53/SH3BGRL-mediated CIC formation [6], it is not likely that CIC formation is regulating chemoresistance through autophagy in mutant p53 or SH3BGRL expressing cells [34, 37, 44].

Finally, mutant p53 cells grow well in anchorage-independent conditions. Normal cells lack the capacity to grow in such conditions as binding to an extracellular matrix is needed for proliferation and cell growth. The result is anoikis, a programmed form of cell death that is the result of loss of binding to surrounding matrix [45]. CIC formation and especially entosis are more prone to occur when cells are detached or rounded up [12]. We see that SH3BGRL promotes the ability of mutant p53 cells and p53 KO cells to grow in anchorage-independent conditions and loss of SH3BGRL reduces colony formation. These data suggest that mutant p53 could regulate SH3BGRL to promote this capacity. Anchorage independent growth capacity might be regulated by CIC formation, but this is technically very hard to determine, but if it is happening likely independent of SH3BGRL expression.

In conclusion, in this manuscript, we reveal that mutant p53 cells drive a process of CIC formation that seems different from entosis and cannibalism and is driven by the host mutant p53 cells that transcriptionally induce *SH3BGRL* expression. CIC formation as well as high *SH3BGRL* expression levels promote etoposide resistance and anchorage-independent growth of mutant p53 cells. As some speculation exists on the role of SH3BGRL as a tumour promoter or suppressor, it will be interesting to investigate this further in the context of p53 status and activity.

## Materials and methods

### Cell culture

A431 (epidermoid carcinoma), SK-BR-3 (Breast adenocarcinoma), and H1299 (non-small cell lung cancer) cell lines were purchased from ATCC (LGC standards). All cell lines were cultured in Dulbecco’s modified eagle’s medium (DMEM) high glucose, pyruvate and glutamine (Gibco, ThermoFisher Scientific) supplemented with 10% foetal bovine serum (FBS) (Sigma-Aldrich, Merck Lifesciences) at 37 °C under 5% CO_2_. Cell lines were mycoplasma tested, confirmed to be contamination free and confirmed for STR markers using Eurofins.

Stable A431 CRISPR p53 KO and mutant p53 Ctrl cell lines were generated using pre-made GFP CRISPR constructs against p53 (TCCATTGCTTGGGACGGCAAGG) or with empty vector (GACTGCTTGTAGATGGCCA) (Sigma-Aldrich, Merck Lifesciences) and lipofectamine-2000 transfection Reagent (ThermoFisher scientific) with OptiMEM I Reduced Serum Medium (ThermoFisher scientific) as described in Mackay et al. [6]. Fluorescent A431 mutp53 and p53 KO cell lines were subsequently established from those cells by transfecting with GFP or mCherry (2 μg/well in 6-well) (Clontech) followed by selection with 600 μg/ml gentamycin (Sigma-Aldrich, Merck Lifesciences) and FACS. A431 mutp53 and p53 KO GFP-SH3BGRL stable cell lines were generated by transfecting cells with human SH3BGRL gene (2 μg/well in 6-well) (Sino Biological) using reverse transfection with lipofectamine 3000 reagent (6 μl/well in 6-well) with enhancer P3000 reagent (2 μl/μg) (ThermoFisher scientific) and subsequent selection with hygromycin (Gibco, ThermoFisher scientific).

### Transient gene overexpression or knockdown and plasmids

For transient gene overexpression, cells were reverse transfected with plasmid DNAs (2 μg/well in 6-well or 5 μg/dish in 10 cm) using lipofectamine 3000 reagent (6 μl/well in 6-well or 18 μl/dish in 10 cm) (ThermoFisher Scientific) and P3000 enhancer reagent (2 μl/μg of DNA) (ThermoFisher scientific) in serum free DMEM media (250 μl/well in 6-well or 500 μl/dish in 10 cm). The Mutp53 175H and 273H plasmid constructs are presented in [6]. The WT p53 plasmid was a gift from Xin Lu and additional p53 mutations were generated using mutagenesis (G105C, L130V, V157F, R159P, V173L, W146D, M246A, M246V, M246I, W146E, 293D, S106R, A159P, M160I, P190L and T230S) using forward and reverse primers listed in Supplementary Table 3. For gene overexpression, GFP-SH3BGRL (2 μg/well) with pCMV3-C-GFPSpark plasmid backbone (HG16804-ACG) was purchased from Sino Biological. Golgi-mEGFP (Golgi-mTurquoise plasmid) (182877), mEGFP-RhoA-C1 (EGFP(A206K)-C1 backbone) (29674) and EGFR-GFP (pEGFP-N1 backbone) (32751) were purchased from Addgene.

For transient gene knockdown, reverse transfection of cells with siRNA (2.5 μl of 20 μM/well in 6-well) and lipofectamine 3000 reagent (6μl/well in 6-well) in serum-free DMEM media (250 μl/well in 6-well) was done. For p53 knockdown, p53 siRNA with fwd: 5′ GACUCCAGUGGUAAUCUAC 3′ and rev: ′3 GUAGAUUACCACUGGAGUC ′5 (Eurofins genomics) was used. For SH3BGRL siRNA (20 μM) transfection, a SMART pool siRNA complex (Dharmacon) with: GCUAUAUUACUGCGAUUAA (siRNA J-019584-17), CCAAUGAAGAGAAUCGGAA (siRNA J-019584-18), CUGUAUACACCAAUGAUUU (siRNA J-019584-19) and AAACAACUCACAAUCGUAA (siRNA J-019584-20) were incorporated. Non-targeting Ctrl siRNA (UGGUUUACAUGUCGACUAA; D-001810-01-05) was purchased from Dharmacon.

### CIC detection in fixed A431 cell co-cultures

For CIC detection, fluorescent A431 cell co-cultures were seeded simultaneously (3 × 10^4^ cells/cell line in 96-well, 2 × 10^5^ cells/cell line in 24-well, 7 × 10^5^ cells/cell line in 35 mm dish) and incubated for 24 h. CIC quantification was manually scored using confocal fluorescent microscopy by considering the following three criteria: 1. Host cells to have a crescent-shaped nucleus. 2. Internal cells to be enclosed within the host vacuole and 3. both cell nuclei need to be detected in the same focal plane. CIC structures were imaged using opera phenix (PerkinElmer) or Zeiss LSM800 confocal (Zeiss Ltd) microscopes, in which multiple fields were selected to be imaged at 20× magnification using 488 nm (for GFP), 594 nm (for mCherry) and 405 nm (for Hoechst) channels. CICs were scored manually from a total number of fields and normalised to the total number of cells indicated by number of Hoechst-positive cells using Harmony software (PerkinElmer) or Fiji imageJ2 software (version 2.14). CIC numbers of key experiments were blindly scored and validated by an independent researcher.

### Immunofluorescence and cell staining

Cell tracker green dye (ThermoFisher Scientific) in DMSO was used at 10 μM and cell tracker red dye (ThermoFisher Scientific) in DMSO was used at 4.21 μM to stain the cells. Cells were incubated with cell tracker red or green dye for 45–60 min at 37 °C (5% CO_2_) followed by washing of the cells with PBS and subsequent co-culturing.

For immunofluorescence, cells were fixed in 4% paraformaldehyde (10 min, 4 °C) (ThermoFisher scientific) followed by permeabilization with 0.1% Triton X-100 for 10 min at 4 °C. (Santa Cruz biotechnology). The cells were then blocked with 2% bovine serum albumin (BSA) (Merck life Science) for 1 h RT and subsequently incubated with primary Ab in 2% BSA with 5% donkey serum (Abcam) at RT for 1 h or at 4 °C for overnight. After that, cells were washed three times with PBS and incubated with secondary Ab in 2% BSA for 1 h at RT. Fixed cells were mounted using vectashield mounting medium (+DAPI) (Abcam).

### Live time-series imaging

Live imaging of cells was performed on the Andor spinning disk microscope (Oxford Instruments) or Incucyte (Sartorius). For the spinning disk, cells were seeded in a 35 mm glass-bottom dish at least 2 h prior to imaging. Timeseries experiments were done with a 20× lens with intervals of 10 min for 24–48 h (37 °C, 5% CO_2_). 488 nm, 594 nm and brightfield channels were used. Images were analysed and produced using Imaris image analysis software (version 9.2) (Oxford Instruments). For the incucyte, cells were counted and co-cultured in 96-well plates for up to 72 h and fluorescent images taken every 4 h.

### Imaging flow cytometry (Image-stream), flow cytometry and FACS of CICs

GFP and mCherry expressing cells were co-cultured (8 × 10^5^–1 × 10^6^ cells/cell line) in 6 cm or 10 cm dishes for 24 h. From the co-cultures, double-positive populations expressing both GFP and mCherry were considered to be CICs. Imagestream files were generated in a rif format, compensated using single colour controls for GFP, mCherry and Hoechst (as a nuclear counterstain) into cif files and subsequently analysed as daf files. Data was analysed using IDEAS v6.2 software (Cytek biosciences). For sorting of pure CIC populations from GFP and mCherry labelled A431 cell co-cultures, gating strategies validated on Imagestream were applied on The Melody^TM^ (BD Biosciences).

### RNA extraction, cDNA synthesis and real-time quantitative PCR

Cell lysis and RNA isolation were done using Norgen’s total RNA purification kit (#17200, Norgen Biotek). RNA sample concentrations and purity were measured on the nanodrop (Labtech International). For cDNA preparation, 1 μg of purified RNA samples was reverse transcribed into cDNAs using the Applied Biosystems high-capacity cDNA reverse transcription (RT) kit (ThermoFisher Scientific) in PTC-200 Peltier thermal cycler (25 °C-10 min, 37 °C, 120 min, 85 °C-5 min and 4 °C-overnight) (MJ Research). qRT- PCR was done by using Applied biosystems power up SYBR green Master Mix kit (ThermoFisher Scientific) on a QuantStudio^TM^ 3 Real-Time PCR System (ThermoFisher scientific) and CFX connect-real-time PCR detection system (Biorad). 500 nM of specific gene oligos (Eurofins Genomics) were added to the cDNA master mixes. Oligos were designed using primer-BLAST tool (National Institute of Health). p53 (fwd): GAGGTTGGCTCTGACTGTAC and p53 (rev): CCGTCCCAGTAGATTACCAC, SH3BGRL (fwd): CTGGCTCTACAGCGATTAAGAA and SH3BGRL (rev): TGGCTGGTCGACTATTTTCAGL and GAPDH (fwd): GCAGAGATGATGACCCTTTTGGCT and GAPDH (rev): TGAAGCTCGGAGTCAACGGATTGGT used.

### Next-generation RNA sequencing

RNA was isolated as described above and for RNA sequencing, indexed PolyA RNA-Seq libraries were prepared using 200 ng of total RNA and 14 cycles of amplification with the Agilent SureSelect Strand Specific RNA Library Prep Kit for Illumina Sequencing (Agilent Technologies, Inc., Cat No: G9691A). Libraries were quality checked by Bioanalyzer (Agilent) and quantified by qPCR using a Kapa Library Quantification Kit (Kapa Biosystems Inc., Cat No: KK4835). Paired-end 75 bp sequencing was carried out by clustering 1.8 pM of the pooled libraries on a NextSeq 500 sequencer (Illumina Inc.)

RNA-Seq reads were quality checked by FASTQC programme (248) and aligned in paired-end mode to the human genome assembly (GRCh38) using the STAR aligner v.2.5.1b (249) with the default parameter setting. Mapped data were converted to gene-level integer read counts by featureCounts (250) using the Ensemble gene annotation (Homo_sapiens.GRCh38.85.gtf). Differential gene expression (DGE) was evaluated comparing the gene-level integer read count data for the knockdown and control samples using the DESeq2 Bioconductor package (251). For DESeq2 DGE estimation, a normalisation factor to model read counts to account for sequencing depth was incorporated. Then, gene-wise dispersions were estimated, and shrinkage analysis was analysed to generate more accurate estimates of dispersion to model the counts. Finally, using the negative binomial model, hypothesis testing was performed using the Wald test. The resulting p-values were adjusted using the Benjamini and Hochberg approach for controlling the false discovery rate (FDR). Genes with an adjusted *p*-value ≤0.05 (FDR ≤ 0.05) between two groups were considered to be differentially expressed. We utilized ClusterProfiler for our enrichment analysis. Our input consists of all differentially expressed genes (DEGs) that exhibit a minimum two-fold change, including both upregulated and downregulated genes.

### Antibodies and immunoblotting

Antibodies used for immunoblotting were: anti-p53 (DO-1) (1:2000, sc-126; Santa Cruz), anti-p53 (FL-393) (1:2000, BS-8687R,Bioss), anti-GFP (1:2000, 6556,Abcam) and anti-GAPDH (1:2000, 60004-1, Protein tech). Cells were lysed using a 1:1 mixture of RIPA (ThermoFisher scientific) and Nonidet P-40 (1% NP40, 150 mM NaCl (ThermoFisher scientific), 100 mM Tris-HCL at pH 8 (Santa Cruz Biotechnology)) with protease inhibitor (PI) (Roche diagnostics) and phosphatase inhibitor tablet (Roche diagnostics) on ice. Lysates were spun at 15,700 × *g* at 4 °C (Eppendorf Ltd) for 15 min. Supernatants were collected, mixed with 4X sample buffer (1 M Tris pH 6.8, 20% SDS, 20% glycerol, 10% 2-Mercaptoethanol, orange G) (Merck Life Sciences) and boiled at 95 °C for 5 min. Sample lysates (30 μl/well) were run (20X MOPS (3-(N-morpholino) propane sulfonic acid) SDS running buffer diluted 1:20 in distilled H_2_O) on 8–10% resolving gels at 100 V for 1 h. SDS-PAGE, protein transfer was done by using a 0.5 mm nitrocellulose membrane (GE Healthcare Lifesciences). Electrophoretic transfer of the purified proteins was done at 200 mA for 1.5–2 h in transfer buffer (100 ml 10X Tris-glycine, 100 ml methanol, 1.8 L distil H_2_O and 10 ml of 10% SDS). Membranes were blocked for 1 h in 5% skimmed milk or 5% bovine serum albumin (BSA) in TBS-tween (Merck Life Sciences). Secondary antibodies IRDye secondary antibodies 680RD or 800CW (Li-Cor, 0.05 µg/ml) were used to visualise expression. Protein bands were detected on an Odyssey Sa infrared imaging system (LI-COR Biosciences UK Ltd).

### Resazurin cell survival assay

For survival assays, cells (1000/well) were seeded in a 96-well plate and incubated overnight (37 °C, 5% CO_2_). After 24 h, etoposide (170 mM) (Merck Life Sciences) concentrations in µM: 0.6, 1.2, 1.7, 1.8, 2.4 (Fig. 5D, F) and 2.5, 3.4, 4.25, 5.66 or 6.8 (Fig. 5B) were added for 72 h. After drug incubation, media were removed and resazurin at 44 µM (Merck Life Sciences) was added to the wells for ~3 h until a media colour change from blue/purple to pink was observed. The plate was read by a spectrophotometer (BioTek Instruments) to measure the flourescence, excitation 520 nm, emission 580–640 nm. Survival in the absence of etoposide was set to 100%.

### Soft-agar colony formation assay

To a 6-well plate, 2 ml/well of 0.5% agar (Merck Life Sciences) was added as the bottom agar layer and allowed to set at RT for at least 30 min. A431 cells were trypsinized and counted. Then, 750 μl/well of cell suspension (2 × 10^4^ cells/well) was mixed with 750 μl/well of 0.8% agar for a 0.4% top layer agar. After that, 1.5 ml/well of cell suspension and agar mixes were added to the wells and incubated overnight (37 °C, 5% CO_2_). On day 5, 500 μl/well of DMEM media was added to the wells. Cells were incubated for 14 days and stained with 0.025% crystal violet for 15 min at RT. The plate was imaged on the Licor Odyssey Sa infrared imaging system at 700 nm. Colonies were counted by analysing particles (size:0–5) from binarized images using Fiji imageJ2 software (version 2.14).

### Tumour xenograft studies

Animal work was done under project licence PP8383164. For the study, six female NSG (NOD SCID gamma) mice (Charles River UK Ltd) were included aged between 7 and 12 weeks old and weighing over 20 g. They were housed in the licensed establishment, University of Durham’s Life Science Support Unit (LSSU) three weeks before the experiment to allow acclimatisation and randomly assigned to study groups by the technical staff of the animal house. Three were subcutaneously injected with A431 mCherry mutp53 + GFP p53 KO cells and the other three with A431 mCherry p53 KO + GFP p53 KO cells. Grouping of mice was done randomly in two different cages with food and water ad libitum on a 12 h dark/light schedule. For both groups, 6 × 10^5^ cells (3 × 10^5^ of each cell line) in 100 μl (PBS + Geltrex 1:1) were injected in each mouse. After injection, tumour burden and mice weight were monitored twice a week and tumour size measured with calliper once tumour formation could be seen. The study was continued until tumour sizes were up to 800 mm^3^ or more than 10% weight loss was observed in the mice or visible signs of distress were evident. Mice were culled using a schedule I cervical dislocation as per ASPA guidelines. Tumour volume was estimated by using the formula(length x width^2^)/2. Tumours were dissected at study end and fixed in 4% PFA for 48 h (4 °C) and then transferred into 70% ethanol (Fisher Scientific) for immunohistochemistry (IHC) staining.

### H&E and Immunohistochemistry (p53)

Sections of tumour xenografts were stained for H&E and p53. For p53, slides were stained on the Ventana Discovery Ultra. 4μm sections of FFPE tumours were cut and mounted on charged slides. For Ki67, dewaxing and heat-induced epitope retrieval of slides was automated on the Bond RX, using Epitope Retrieval Solution 1 (ER1) (Leica Microsystems, AR9961) for 20 min at 98 °C. Using the Refine kit (Leica Microsystems, DS9800), endogenous peroxidase was blocked using the Peroxidase in the Refine kit for 10 min and the slides further blocked with 10% w/v casein (Vector, SP5020) in TBST. For p53, dewaxing and heat-induced epitope retrieval of slides was automated on the Discovery Ultra using the antigen retrieval solution CC1 (5424569001) for 36 min at 100 °C. Anti-p53 (M7001; 237 mg/ml) staining was done for 32 min at 37 °C followed by detection using the Ultraview kit (5269806001). Slides were dehydrated through graded ethanol, cleared in Xylene and coverslipped with Pertex (Cell Path SEA-0100-00A). Samples were scanned and viewed using Olympus vs200 ASW 3.2.1 (Build 21655) (Olympus Life sciences). CIC was quantified from nine random fields in one H&E-stained whole tissue section of each co-culture condition (Ctrl (mutp53)+ p53 KO or p53 KO+ p53 KO) using OlyVIA software (Olympus Life Sciences). Slides were randomly labelled by the histology service (blinded for the researchers) and CIC numbers counted.

### Retrospective analyses of TCGA, TARGET and GTEx datasets

The Cancer Genome Atlas (TCGA), Therapeutically Applicable Research to Generate Effective Treatments (TARGET) and non-cancer Genotype Tissue Expression (GTEx) datasets were used for *SH3BGRL* expression and survival analyses in database patient samples. For comparison of gene expression (RNA seq) across different datasets, the “TCGA TARGET GTEx” study was incorporated. Within TCGA datasets based on cancer type, *TP53* mutation status and *SH3BGRL* gene expression level were chosen as ‘genomic variables’ to stratify the patients. Analysis of *SH3BGRL* gene expression levels in different tissue sites (primary tumour, normal tissues and metastatic sites) was done by grouping based on ‘phenotypic variable’ of sample type within TCGA dataset. Survival data was extracted to generate Kaplan–Meier survival curves using Graphpad Prism.

### Uptake of beads or fixed cells

5 μl/well of carboxylate-modified polystyrene fluorescent beads (Merck Life Sciences) were added to cells after thorough homogenisation. Cells were incubated with beads for 24 h (37 °C, 5% CO_2_). Then, cells were washed 3X with PBS, trypsinized and spun (1150 g, 5 min). Cell pellets were later resuspended in 2 ml/sample of PBS for flow cytometry analysis. To add fixed cells for uptake assays, 3% glyoxal (Merck Life Sciences) was used as the fixing agent. Fixation of GFP p53 KO cells or mCherry Ctrl (mutp53) cells was done at 4 °C for 20 min. Fixed cells were resuspended in media and stored at 4 °C prior to being added on live cells.

### Statistical analyses

Prism 10 (GraphPad Prism 10.2.3 (347); GraphPad software) was used for all graphing, statistical tests and analyses. Samples were assumed to follow a Gaussian (normal) distribution as for most, *n* = 3 sample sizes often show up as ‘n too small’ for normality tests. For paired groups, parametric two-tailed unpaired student’s *t*-test was used. For comparison of more than two groups with one independent variable, ordinary one-way ANOVA was incorporated. On the other hand, analyses of two independent variables from more than two groups was done by two-way ANOVA (Corrected for multiple comparisons using Šídák test). Analyses of etoposide dose dependent effects on cell survival (%) was done through nonlinear regression models (inhibitor vs normalized response). Evaluation of Kaplan–Meier curves was done to determine patients’ overall survival. Comparison of Kaplan–Meier curves was done by applying both Log-rank (Mantel–Cox) test and Gehan–Breslow–Wilcoxon test.

## Supplementary information

Supplemental Figure legends

Supplemental Figures

Supplemental Table 1

Supplemental Table 2

Supplemental Table 3

original data

Supplemental Video

## Data Availability

We deposited our NGS data in the GEO repository under accession number GSE293711.
