# Supplementary material for: Mutant p53 induces SH3BGRL expression to promote cell engulfment
Source: Cell Death Discov. 2025 Jul 1;11:288. doi: 10.1038/s41420-025-02582-x (PMC12218370; doi:10.1038/s41420-025-02582-x)
Supplement: Supplementary file 1 — Supplemental Figure legends [file 41420_2025_2582_MOESM1_ESM.docx]

**Supplemental Fig. 1**

A and B) CIC quantification in A431 GFP Ctrl (mutp53) + mCherry p53 KO cell co-cultures seeded at different cell densities and measured at different time points (n=3, Error bars=SD). B is corrected for total cell number. C) Schematic of experimental setup for D. D) (Left) Number of CIC in 9 fields of tissue sections derived from co-cultures of A431 Ctrl (mutp53) and A431 p53 KO cells that were xenografted subcutaneously in mice (student’s t-test, *=p≤0.05, Error bars=SD). CIC was quantified from histological sections. (Right) Histological sections of xenografted co-cultures of A431 Ctrl (mutp53) with p53 KO cells or p53 KO with p53 KO cells stained for p53(Scale bars=50μm).E) Schematic of experimental setup for co-cultures used in F-I. F) A431 Ctrl (mutp53) cells were transfected with siRNA targeting p53 (p53si) or a control (Ctrlsi) and labelled with cell tracker red (orange) or green dyes. The cells were co-cultured for 24 hrs. CIC was quantified in confocal images using fluorescence to detect cell status (n=3, two-way ANOVA, **=p≤0.01, ****=p≤0.0001, Error bars=SD). G) Examples of confocal images of co-cultures. Blue staining is Hoechst and scale bars indicate 20μm. H-I) Knockdown of p53 was validated in western blot (H) and bands quantified using the Li-Cor (I) (; n=3, two-way ANOVA, ***=p≤0.001, ****=p≤0.0001, Error bars=SD). GAPDH was used as loading control.

**Supplemental Fig. 2**

A) RhoA expression as measured by western blot using GFP to detect overexpressed RhoA and RhoA to detect endogenous levels. GAPDH was used as loading control B) Schematic of experimental setup for C. C) Quantification of CIC structures in Ctrl (mutp53) and p53 KO cells transfected with GFP-RhoA. CICs consisting of GFP positive cells were counted and host or inner cell status scored (n=3, two-way ANOVA, *=p≤0.05, Error bars = SD). D) CIC quantification in live A431 mCherry (Ch) Ctrl (mutp53) + GFP p53 KO cells (live or fixed) and in live GFP p53 KO + mCherry (Ch) Ctrl (mutp53) (live or fixed) cell co-cultures (n=3, two-way ANOVA, **=p≤0.01, Error bars=SD). NB. This graph is combining data of Fig. 1D and 2F allowing a direct comparison. E) GO enrichment of pathways identified more frequently in A431 p53 KO cells compared to both A431 parental (mutp53) cells and Ctrl (mutp53) cells. F) mRNA frequencies of *RSAD2* and *VGLL1* in A431 Ctrl (mutp53) cells with CtrlsiRNA or p53siRNA conditions (n=3, two-way ANOVA, **=p≤0.01, Error bars=SD).

**Supplemental Fig. 3**

A) Overall survival of all cancer patients with or without TP53 mutations using the TCGA Pan Cancer database (Log rank mantel cox test). B) Mean *SH3BGRL* Log_2_ fold change expression in cancer patients or non-diseased individuals from the TCGA TARGET GTEx database (student’s t-test, ****=p≤0.0001, Error bars=SD). C)Overall survival of cancer patients with high or low *SH3BGRL* expression using the TCGA PanCan database (Log rank mantel cox test). D) Overall survival of patients across all cancer (Pan-can) stratified into ones with TP53 mutation and high or low *SH3BGRL* expression, respectively and with WTp53 with high or low *SH3BGRL* expression, respectively (Log rank mantel cox test, ****=p≤0.0001). E) Mean *SH3BGRL* log_2_ fold change in carcinoma and non-diseased epithelial tissues (TCGA and GTEx, student’s t-test, *=p≤0.05, Error bars=SD). F) Overall survival of TCGA patients with carcinoma grouped into those with TP53 mutation or WTp53 coupled with high or low SH3BGRL expression, respectively (Log rank mantel cox test, *** = p≤0.001, ****=p≤0.0001). G) SH3BGRL log_2_ fold changes in breast invasive carcinoma and non-diseased breast tissues (TCGA and GTEx, student’s t-test, ****=p≤0.0001, Error bars=SD). H) Overall survival of TCGA breast invasive carcinoma patients with TP53 mutation or WTp53 along with high or low SH3BGRL expression, respectively (Log rank mantel cox test).

**Supplementary Fig. 4**

A) qRT-PCR validation of SH3BGRL knockdown in mCherry or GFP labelled A431 Ctrl (mutp53) cells used for CIC quantification in 4B (n=3, one-way ANOVA, **=p≤0.01, Error bars=SD).

**Supplemental Fig. 5**

A) Survival curves corresponding to Fig. 5A. A431 Ctrl (mutp53) or p53 KO cells stably expressing GFP-SH3BGRL or GFP were grown in resazurin survival assays and treated with increasing doses of etoposide. B-C) A431 mCherry Ctrl (mutp53) and GFP p53 KO cells were co-cultured. Single (B) and focused (C) cells were first selected using imagestream flow cytometry. D) mCherry and GFP fluorescence were applied to detect double positive CIC populations (positive for both mCherry and GFP fluorescence). E) Representative image of a host mCherry Ctrl (mutp53) cell with an internalised GFP p53 KO cell. F) CIC populations were further selected for single and rounded cells to exclude double positive cell clusters or adjacent cells based on their area and major axis (wideness of cells). G) From single and rounded CIC populations, true ‘CIC’ populations were validated through visual detection of the structures. Ones with external red mCherry Ctrl (mutp53) cells carrying internal GFP p53 KO cells (96%) and others with external GFP p53 KO cells carrying internalised mCherry Ctrl (mutp53) cells (4%) were quantified. H) 82% of CICs detected through imagestream were single and rounded and from those, 90.85% were true CICs. This validates the reliable ability of flow cytometry and FACs in detecting and sorting CIC populations from fluorescent cell co-cultures. I) CIC quantification in FACS sorted double positive CIC-derived or in non FACS sorted co-cultures of A431 mCherry Ctrl (mutp53) + GFP p53 KO cells (n=3, two-way ANOVA, Error bars=SD).

**Supplemental Movie 1**

Video showing the uptake of bead (5μm) in A431 Ctrl (mutp53) cells using spinning

disk confocal microscope (Scale bar=5μm).

**Supplemental Table 1** Mutation frequencies of selected p53 mutants in all cancer

**Supplemental Table 2** List of genes differentially expressed in A431 p53 KO cells relative to that in A431 parental and Ctrl (mutp53) cells

**Supplemental Table 3** qRT-PCR oligo sequences of p53 mutants
