# Supplementary material for: Mutant p53 induces SH3BGRL expression to promote cell engulfment
Source: Cell Death Discov. 2025 Jul 1;11:288. doi: 10.1038/s41420-025-02582-x (PMC12218370; doi:10.1038/s41420-025-02582-x)

Supplemental Figure 1

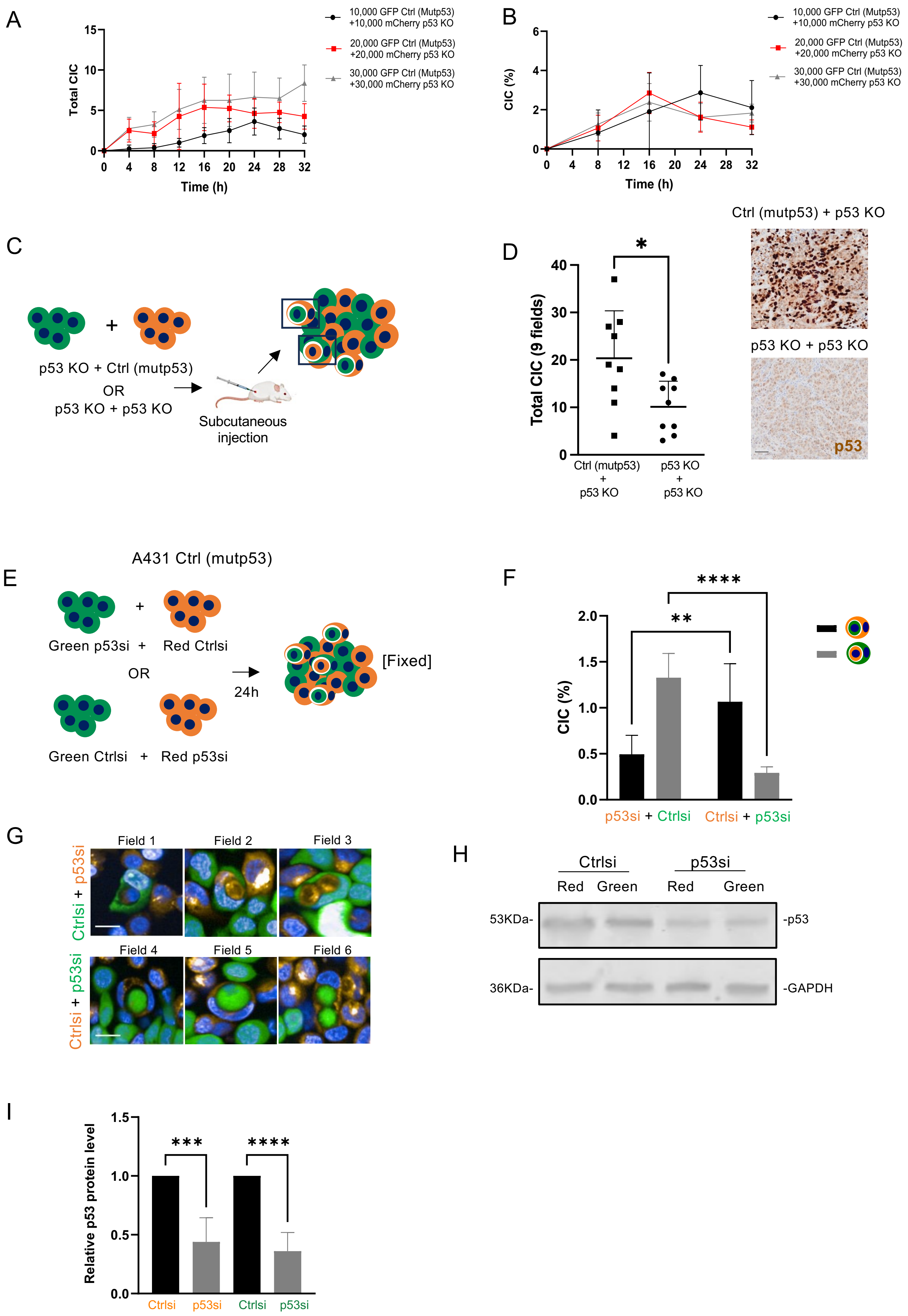

Supplemental Figure 2

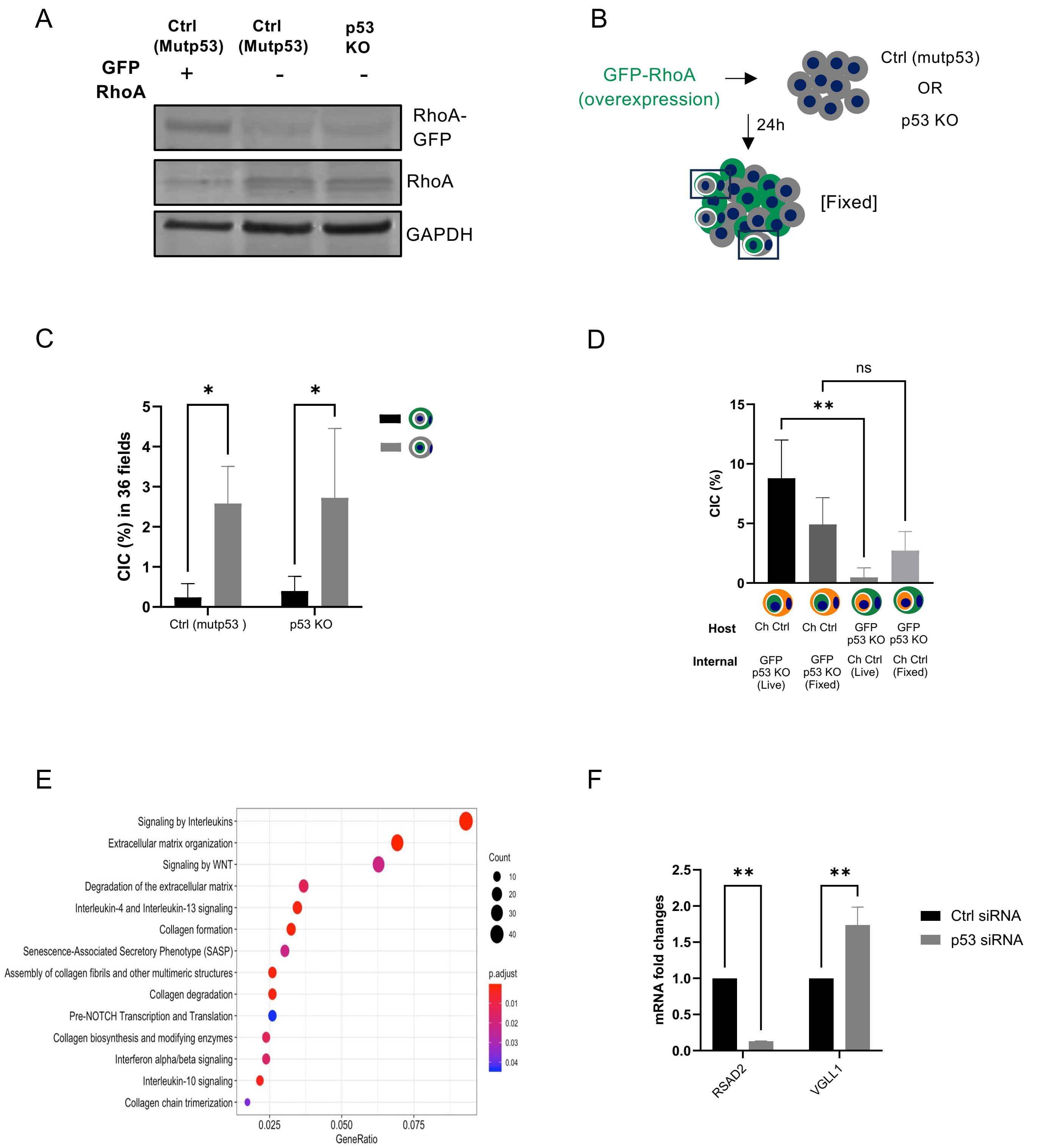

Supplemental Figure 3

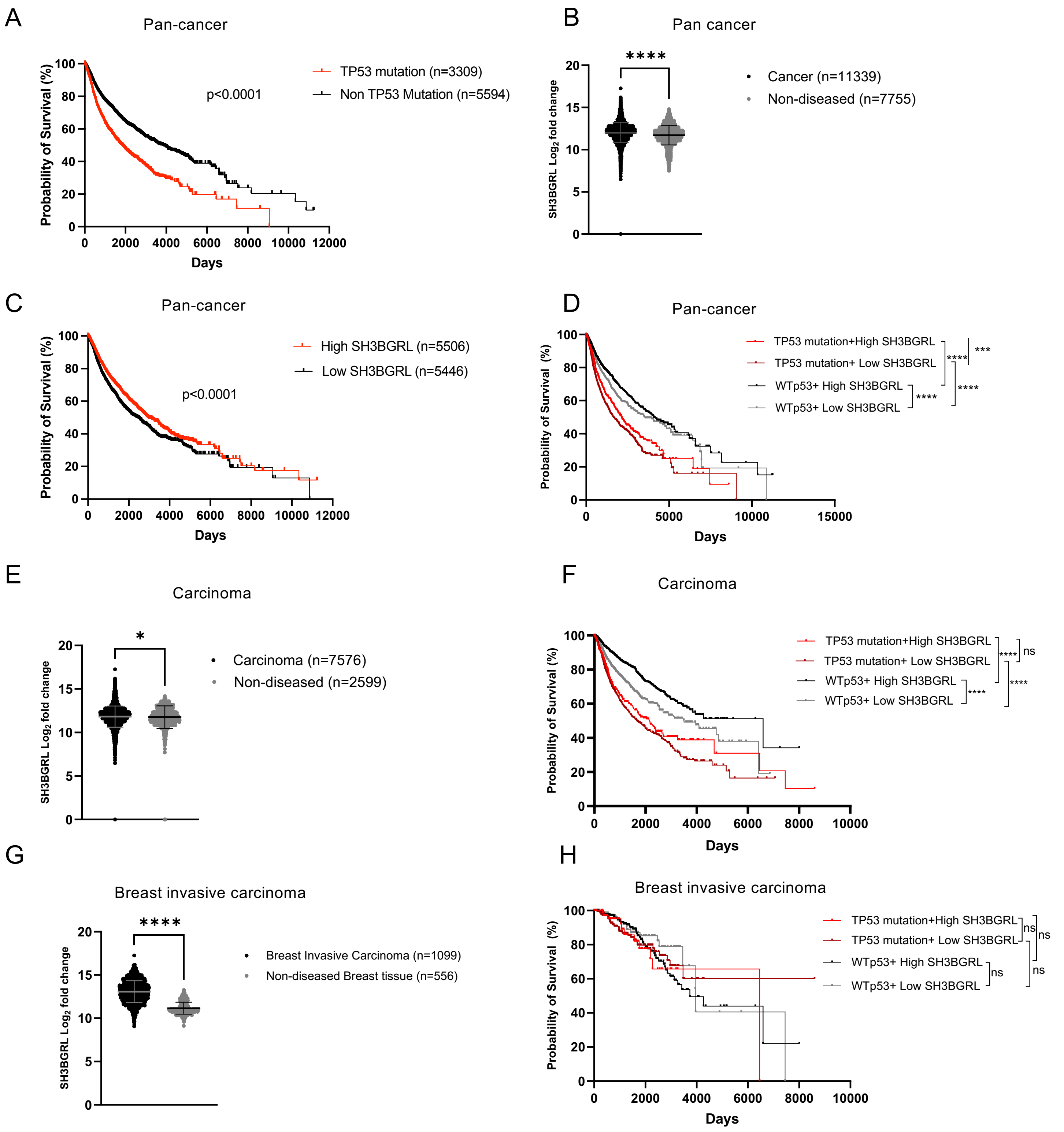

Supplemental Figure 4

A

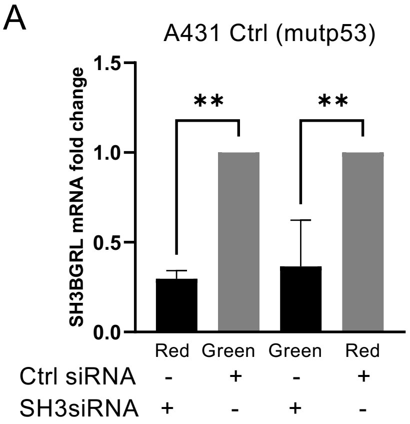

Supplemental Figure 5

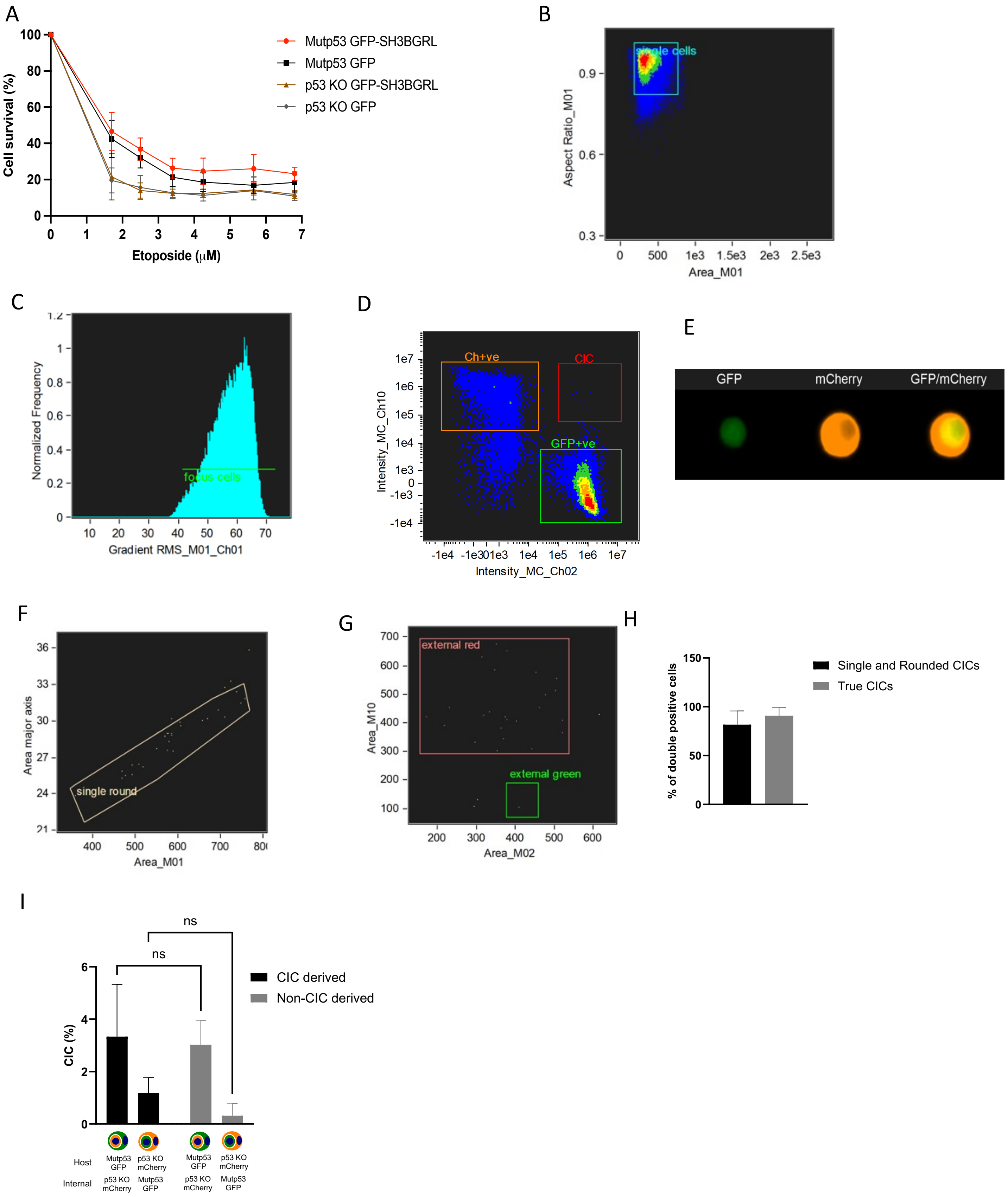

Supplement: Supplementary file 2 — Supplemental Figures [file 41420_2025_2582_MOESM2_ESM.pdf]
