# Supplementary material for: Mutant p53 induces SH3BGRL expression to promote cell engulfment
Source: Cell Death Discov. 2025 Jul 1;11:288. doi: 10.1038/s41420-025-02582-x (PMC12218370; doi:10.1038/s41420-025-02582-x)
Supplement: Supplementary file 3 — Supplemental Table 1 [file 41420_2025_2582_MOESM3_ESM.docx]

**Supplemental Table 1 TP53 Mutation frequency in all cancers**

| **Codon** | **AA change** | **Frequency** | **Mutation** | **Type** |
| --- | --- | --- | --- | --- |
| 105 | G105C | 0.013382 | 313G>T | NF |
|  |  | 0 | 313-315delinsTGT | NF |
| 130 | L130V | 0.076946 | 388C>G | NF |
|  |  | 0 | 388-390 delinsGTG | NF |
| 157 | V157F | 0 | 468-469delinsAT | NF |
|  |  | 0.006691 | 468-469delinsGT | NF |
|  |  | 0.003345 | 468-469delinsTTT | NF |
|  |  | 0.702553 | 469G>T | NF |
| 159 | A159S | 0.030109 | 475G>T | PF |
|  |  | 0.003345 | 475-476delinsAG | PF |
| 173 | V173L | 0.230839 | 517G>T | NF |
|  |  | 0.073601 | 517G>C | NF |
|  |  | 0 | 517-519deliinsTTA | NF |
| 175 | R175H | 4.068114 | 524G>A | NF |
|  |  | 0 | 524-525delinsAT | NF |
| 146 | W146D | 0 | 436-438delinsGAC | NF |
| 246 | M246A | 0 | 736-737delinsGC | NA |
|  |  | 0 | 736-738delinsGCC | NA |
|  | M246V | 0.204075 | 736A>G | NF |
|  | M246I | 0.120438 | 738G>A | NF |
|  |  | 0.016727 | 738G>C | NF |
|  |  | 0.020073 | 738G>T | NF |
| 273 | R273H | 2.870429 | 818G>A | NF |
|  |  |  | 818-819delinsAC | NF |
| 106 | S106R | 0 | 316A>C | PF |
|  |  | 0 | 318C>A | PF |
|  |  | 0.023418 | 318C>G | PF |
| 146 | W146E | 0 | 436-437delinsGAC | NA |
| 159 | A159P | 0.10371 | 475G>C | NF |
| 160 | M160I | 0.030109 | 480G>A | PF |
|  |  | 0.010036 | 480G>T | PF |
|  |  | 0.013382 | 480G>C | PF |
| 190 | P190L | 0.173965 | 569C>T | PF |
|  |  | 0 | 569-570delinsTG | PF |
| 230 | T230S | 0.003345 | 688A>T | PF |
|  |  | 0 | 689C>G | PF |
| 293 | G293D | 0 | 878-879 delinsAC | NA |

Mutation frequency in all cancers using the TP53 database. Annotated are the codon, the amino acid (AA) change, frequency in all cancers (total known 29891 mutations), the change on the DNA and whether or not a mutation causes a non-functional (NF), partially functional (PF) or a p53 mutant with unknown function (NA).
