# Supplementary material for: Mutant p53 induces SH3BGRL expression to promote cell engulfment
Source: Cell Death Discov. 2025 Jul 1;11:288. doi: 10.1038/s41420-025-02582-x (PMC12218370; doi:10.1038/s41420-025-02582-x)
Supplement: Supplementary file 4 — Supplemental Table 2 [file 41420_2025_2582_MOESM4_ESM.docx]

**Supplemental Table 2** Differentially expressed genes in A431 p53 KO cells relative to that in A431 parental (mutp53) and Ctrl (mutp53) cells with in red the genes validated in figure 3C.

| **Upregulated genes in p53 KO** | **Log2 FoldChange** | **p value** | **Downregulated genes in p53 KO** | **Log2 FoldChange** | **p value** |
| --- | --- | --- | --- | --- | --- |
| RSAD2 | 6.21 | 0.00E+00 | GJA1 | -4.57 | 1.75E-105 |
| SLFN11 | 6.01 | 2.13E-66 | SATB1 | -4.21 | 2.15E-56 |
| CEACAM6 | 5.85 | 6.32E-11 | AC010127.3 | -4.01 | 5.28E-18 |
| LIMCH1 | 5.78 | 4.20E-74 | PCDH18 | -3.96 | 6.03E-09 |
| KRT6B | 5.19 | 3.24E-13 | KLF8 | -3.85 | 1.69E-22 |
| NGEF | 4.94 | 2.46E-46 | RP11-43F13.1 | -3.71 | 4.23E-12 |
| CEACAM5 | 4.82 | 5.45E-17 | MANSC1 | -3.65 | 2.13E-29 |
| LINC00431 | 4.73 | 8.51E-09 | EN1 | -3.50 | 3.33E-24 |
| TRIB2 | 4.66 | 2.00E-41 | MCTP1 | -3.43 | 9.97E-12 |
| PTPRR | 4.53 | 5.71E-15 | ARL11 | -3.37 | 1.52E-09 |
| B3GNT7 | 4.48 | 1.01E-08 | ZDHHC1 | -3.32 | 2.17E-19 |
| INHBA | 4.47 | 2.03E-46 | SH3BGRL | -3.25 | 5.90E-48 |
| PLCG2 | 4.44 | 5.45E-77 | CXCR2 | -3.20 | 6.59E-09 |
| SERPINB9 | 4.35 | 6.16E-24 | C1orf21 | -3.19 | 4.48E-22 |
| EDARADD | 4.26 | 6.45E-14 | NECTIN3 | -3.09 | 6.01E-17 |
| PPP4R4 | 4.23 | 7.75E-17 | RP11-19E11.1 | -2.99 | 3.44E-09 |
| B3GALT5 | 4.20 | 2.33E-15 | TMEM64 | -2.97 | 2.50E-19 |
| INPP5D | 4.16 | 4.39E-31 | VGLL1 | -2.93 | 7.54E-74 |
| RASAL1 | 4.14 | 9.45E-23 | GRHL3 | -2.89 | 8.48E-61 |
| SPINK5 | 4.11 | 3.54E-09 | ADGRL2 | -2.85 | 2.13E-12 |
| RPS6KA2 | 3.99 | 2.75E-19 | C2orf54 | -2.79 | 1.16E-15 |
| ANO1 | 3.97 | 0.00E+00 | MAN1A1 | -2.69 | 1.20E-08 |
| CAMK1D | 3.95 | 1.78E-23 | CALML3 | -2.63 | 1.47E-49 |
| RP5-1028K7.2 | 3.93 | 2.51E-10 | SETBP1 | -2.63 | 1.48E-09 |
| DNAJC15 | 3.91 | 2.53E-11 | PLXND1 | -2.49 | 6.50E-65 |
| PRKCQ | 3.87 | 4.51E-11 | TMEM139 | -2.46 | 1.72E-26 |
| TGFB2 | 3.81 | 9.77E-27 | BTC | -2.34 | 8.81E-16 |
| CCL22 | 3.74 | 5.97E-29 | KLHDC8B | -2.28 | 3.94E-26 |

| RP11-588H23.3 | 3.60 | 1.04E-28 | YPEL3 | -2.20 | 9.00E-13 |
| --- | --- | --- | --- | --- | --- |
| MT-TF | 3.55 | 9.49E-14 | GGT6 | -2.19 | 6.45E-43 |
| COL18A1 | 3.42 | 1.74E-57 | BCAM | -2.16 | 1.95E-78 |
| SDK1 | 3.40 | 3.22E-16 | CRIP2 | -2.14 | 3.66E-28 |
| CSF2 | 3.38 | 5.63E-13 | FAM171B | -2.12 | 1.81E-11 |
| SULF2 | 3.37 | 0.00E+00 | ABTB1 | -2.08 | 6.59E-13 |
| CCL5 | 3.36 | 6.72E-12 | TFDP2 | -1.96 | 1.15E-43 |
| LINC00704 | 3.31 | 3.42E-14 | CCDC62 | -1.94 | 2.36E-09 |
| GSDMC | 3.20 | 3.52E-09 | SLC12A7 | -1.93 | 5.27E-29 |
| NKX3-1 | 3.18 | 7.93E-29 | SMPDL3B | -1.92 | 2.06E-13 |
| IL7R | 3.16 | 1.00E-80 | RASGEF1A | -1.90 | 9.74E-14 |
| TLR4 | 3.16 | 9.17E-21 | ALDH3A1 | -1.90 | 4.22E-19 |
| PYCARD | 3.15 | 2.64E-20 | AKAP12 | -1.88 | 1.01E-13 |
| GPNMB | 3.14 | 3.39E-24 | MUC20P1 | -1.88 | 2.56E-22 |
| CREG2 | 3.11 | 3.50E-24 | THNSL2 | -1.83 | 1.50E-11 |
| RP11-119F19.5 | 3.10 | 3.98E-12 | MUC20 | -1.81 | 1.05E-24 |
| OLFML2A | 3.05 | 1.95E-18 | ZNF750 | -1.80 | 1.88E-09 |
| CCL28 | 3.02 | 8.98E-19 | ALDH1L2 | -1.75 | 1.49E-09 |
| CPEB2 | 2.98 | 4.08E-10 | ZNF862 | -1.74 | 9.71E-10 |
| BMP6 | 2.96 | 6.27E-11 | DSEL | -1.73 | 2.39E-13 |
| EMP3 | 2.91 | 3.73E-38 | KIF7 | -1.70 | 1.34E-09 |
| CYP27C1 | 2.86 | 2.18E-23 | OR2A1-AS1 | -1.66 | 3.32E-11 |
| HTR7 | 2.80 | 3.60E-28 | SEMA3F | -1.66 | 7.14E-25 |
| KRT6A | 2.75 | 2.77E-10 | CST6 | -1.64 | 1.58E-12 |
| GNAO1 | 2.74 | 5.88E-19 | CCDC80 | -1.63 | 3.39E-48 |
| SLC16A14 | 2.74 | 1.63E-09 | ANXA9 | -1.62 | 2.42E-16 |
| SPOCK1 | 2.62 | 3.95E-43 | DUOXA1 | -1.61 | 1.01E-19 |
| SLC7A2 | 2.59 | 1.30E-15 | ADPRH | -1.57 | 1.80E-08 |
| ACVR1C | 2.57 | 6.92E-11 | CA2 | -1.57 | 1.10E-37 |
| VNN1 | 2.57 | 9.12E-12 | ACPP | -1.56 | 9.45E-10 |
| NEDD9 | 2.54 | 9.43E-17 | PCDH7 | -1.53 | 9.35E-15 |

| MGAT5B | 2.54 | 4.26E-15 | SEC14L2 | -1.51 | 9.68E-26 |
| --- | --- | --- | --- | --- | --- |
| AASS | 2.51 | 4.80E-12 | EGR1 | -1.51 | 2.79E-34 |
| EIF4E3 | 2.50 | 1.56E-21 |  |  |  |
| VEGFC | 2.46 | 5.86E-90 |  |  |  |
| CALB2 | 2.44 | 9.15E-35 |  |  |  |
| RP11-44F14.8 | 2.41 | 8.76E-10 |  |  |  |
| F2R | 2.39 | 7.82E-33 |  |  |  |
| CLCA2 | 2.38 | 2.32E-09 |  |  |  |
| AC006262.5 | 2.27 | 1.19E-37 |  |  |  |
| NPTXR | 2.27 | 2.75E-09 |  |  |  |
| FSTL3 | 2.24 | 1.25E-21 |  |  |  |
| GBP4 | 2.22 | 3.90E-28 |  |  |  |
| IER3 | 2.21 | 3.38E-58 |  |  |  |
| TNFSF15 | 2.19 | 2.32E-15 |  |  |  |
| ADAMTS12 | 2.18 | 7.11E-36 |  |  |  |
| SERPINA1 | 2.17 | 3.55E-10 |  |  |  |
| ENO2 | 2.16 | 5.15E-37 |  |  |  |
| CASP14 | 2.16 | 8.22E-32 |  |  |  |
| LHFP | 2.14 | 7.34E-21 |  |  |  |
| THBS1 | 2.13 | 2.41E-28 |  |  |  |
| SLIT2 | 2.13 | 4.42E-16 |  |  |  |
| EPSTI1 | 2.13 | 2.54E-16 |  |  |  |
| AHNAK2 | 2.12 | 7.83E-29 |  |  |  |
| RP11-44F14.2 | 2.11 | 1.15E-09 |  |  |  |
| PMEPA1 | 2.09 | 1.49E-37 |  |  |  |
| POF1B | 2.06 | 1.46E-10 |  |  |  |
| SGK1 | 2.06 | 4.58E-19 |  |  |  |
| NRG1 | 2.05 | 3.38E-23 |  |  |  |
| SAA1 | 2.03 | 5.40E-18 |  |  |  |
| TNRC6C | 2.01 | 3.36E-12 |  |  |  |

| ZDHHC2 | 1.98 | 6.44E-41 |
| --- | --- | --- |
| PODXL | 1.95 | 7.61E-34 |
| AOX1 | 1.93 | 1.80E-12 |
| TNS4 | 1.93 | 1.05E-37 |
| PLAT | 1.93 | 7.98E-30 |
| H19 | 1.92 | 4.62E-34 |
| PORCN | 1.89 | 2.43E-37 |
| ZNF117 | 1.88 | 2.31E-11 |
| ZNF365 | 1.81 | 1.30E-10 |
| GALNT12 | 1.80 | 1.95E-32 |
| MT-RNR1 | 1.76 | 5.40E-24 |
| PRSS23 | 1.76 | 1.18E-60 |
| TNFAIP3 | 1.74 | 3.51E-14 |
| TMEM27 | 1.73 | 2.08E-09 |
| HSPA12A | 1.73 | 8.63E-11 |
| DUSP6 | 1.72 | 1.50E-26 |
| FZD1 | 1.72 | 3.68E-15 |
| ST6GALNAC5 | 1.71 | 2.02E-08 |
| GOLGA7B | 1.71 | 1.70E-14 |
| CD24 | 1.71 | 1.75E-69 |
| PIM2 | 1.70 | 4.30E-24 |
| IFIT3 | 1.70 | 1.26E-79 |
| MAPRE2 | 1.70 | 1.27E-46 |
| ZNF114 | 1.70 | 9.49E-35 |
| TNFSF9 | 1.67 | 1.28E-17 |
| ENDOD1 | 1.67 | 1.35E-18 |
| PPP2R2C | 1.66 | 1.61E-56 |
| OAS2 | 1.64 | 2.41E-23 |
| PPP1R14C | 1.64 | 2.39E-54 |
| ANKRD10 | 1.63 | 1.94E-31 |

| CCBE1 | 1.63 | 6.08E-16 |
| --- | --- | --- |
| HSPB1 | 1.60 | 2.19E-16 |
| SEMA6B | 1.60 | 2.64E-31 |
| ACKR3 | 1.60 | 1.23E-15 |
| FAM102B | 1.60 | 1.54E-11 |
| FGF2 | 1.59 | 5.69E-21 |
| NAV1 | 1.59 | 3.22E-20 |
| SH3PXD2A-AS1 | 1.57 | 1.19E-10 |
| MILR1 | 1.57 | 3.58E-13 |
| COL17A1 | 1.57 | 6.49E-47 |
| GPR176 | 1.57 | 4.23E-30 |
| MYRF | 1.56 | 1.95E-09 |
| CYP26B1 | 1.56 | 6.54E-09 |
| A4GALT | 1.55 | 2.28E-10 |
| ALDH1L1 | 1.55 | 2.31E-14 |
| ZNF703 | 1.54 | 6.63E-10 |
| CEACAM19 | 1.53 | 1.76E-09 |
| OASL | 1.53 | 8.97E-34 |
| EPB41L2 | 1.52 | 4.70E-30 |
| NFIX | 1.52 | 3.31E-24 |
| CDC14A | 1.51 | 7.37E-10 |
| PMAIP1 | 1.51 | 1.84E-29 |
| MX2 | 1.50 | 5.12E-16 |
| LRRC8C | 1.50 | 6.45E-15 |
