# Supplementary material for: Mutant p53 induces SH3BGRL expression to promote cell engulfment
Source: Cell Death Discov. 2025 Jul 1;11:288. doi: 10.1038/s41420-025-02582-x (PMC12218370; doi:10.1038/s41420-025-02582-x)
Supplement: Supplementary file 5 — Supplemental Table 3 [file 41420_2025_2582_MOESM5_ESM.docx]

**Supplemental Table 3 qRT PCR oligos of p53 mutants**

| W146P Fwd: ACCTGCCCTGTGCAGCTGCCGGTTGATTCCACACCCCCG  Rev: CGGGGGTGTGGAATCAACCGGCAGCTGCACAGGGCAGGT |
| --- |
|  |
| W146E Fwd: ACCTGCCCTGTGCAGCTGGAGGTTGATTCCACACCCCCG  Rev: CGGGGGTGTGGAATCAACCTCCAGCTGCACAGGGCAGGT |
|  |
| M246E Fwd: AGTTCCTGCATGGGCGGCGAGAACCGGAGGCCCATCCTC  Rev: GAGGATGGGCCTCCGGTTCTCGCCGCCCATGCAGGAACT |
|  |
| M246V Fwd: AGTTCCTGCATGGGCGGCGTGAACCGGAGGCCCATCCTC  Rev: GAGGATGGGCCTCCGGTTCACGCCGCCCATGCAGGAACT |
|  |
| M246I Fwd: AGTTCCTGCATGGGCGGCATTAACCGGAGGCCCATCCTC  Rev: GAGGATGGGCCTCCGGTTAATGCCGCCCATGCAGGAACT |
|  |
| M246A. Fwd: AGTTCCTGCATGGGCGGCGCGAACCGGAGGCCCATCCTC  Rev: GAGGATGGGCCTCCGGTTCGCGCCGCCCATGCAGGAACT |
|  |
| S106R. Fwd: TCCCAGAAAACCTACCAGGGCAGGTACGGTTTCCGTCTGGGCTTC  Rev: GAAGCCCAGACGGAAACCGTACCTGCCCTGGTAGGTTTTCTGGGA |
|  |
| G105C Fwd: CCCTTCCCAGAAACCTACCAGTGCAGCTACGGTTTCCGTCTGGGCTTC  Rev:GAAGCCCAGACGGAAACCGTAGCTGCACTGGTAGGTTTCTGGGAAGGG |
|  |
| P190L Fwd: CTCAGATAGCGATGGTCTGGCCCTTCCTCAGCA  Rev: CCACTCGGATAAGATGCTGAGGAGGGGCCAG |
|  |
| A159P Fwd: CCCGCCCGGCACCCGCGTCCGCCCCATGGCCATCTA  Rev GTGACTGCTTGGTAGATGGCCATGGCGCGGACGC |
|  |
| L130V Fwd: GACTTGCACGTACTCCCCTGCCGTCAACAAGATGTT  Rev: CCAGTTGGCAAAACATCTTGTTGACGGCAGGGGAGT |
|  |
| M160I Fwd: GCCCGGCACCCGCGTCCGCGCCATAGCCATCTA  Rev: GCTGTGACTGCTTGTAGATGGCTATGGCGCGGACGC |
|  |
| T230S Fwd: GCCTGAGGTTGGCTCTGACTGTTCCACCATCCA  Rev ACATGTAGTTGTAGTGGATGGTGGAACAGTCAG |
|  |
| V173L Fwd: GTCACAGCACATGACGGAGGTTCTGAGGCGCTG  Rev GCTCATGGTGGGGGCAGCGCCTCAGAACCTCCG |
|  |
| 293D Fwd: CATCCACTACAACTACATGTGTGACAGTTCCTGCAT  Rev TCATGCCGCCCATGCAGGAACTGTCACACATGT |
|  |
| V157F Fwd: ACACCCCCGCCCGGCACCCGCTTCCGCGCCATGGCCATCTACAAG  Rev CTTGTAGATGGCCATGGCGCGGAAGCGGGTGCCGGGCGGGGGTGT |
|  |
| R159S Fwd: CCCCCGCCCGGCACCCGCGTCCGCTCCATGGCCATCCACAAGCAG  Rev CTGCTTGTAGATGGCCATGGAGCGGACGCGGGTGCCGGGCGGGGG |
