# Supplementary figures and images for: Mutant p53 induces SH3BGRL expression to promote cell engulfment
Source: Cell Death Discov. 2025 Jul 1;11:288. doi: 10.1038/s41420-025-02582-x (PMC12218370; doi:10.1038/s41420-025-02582-x)

Figure 1A

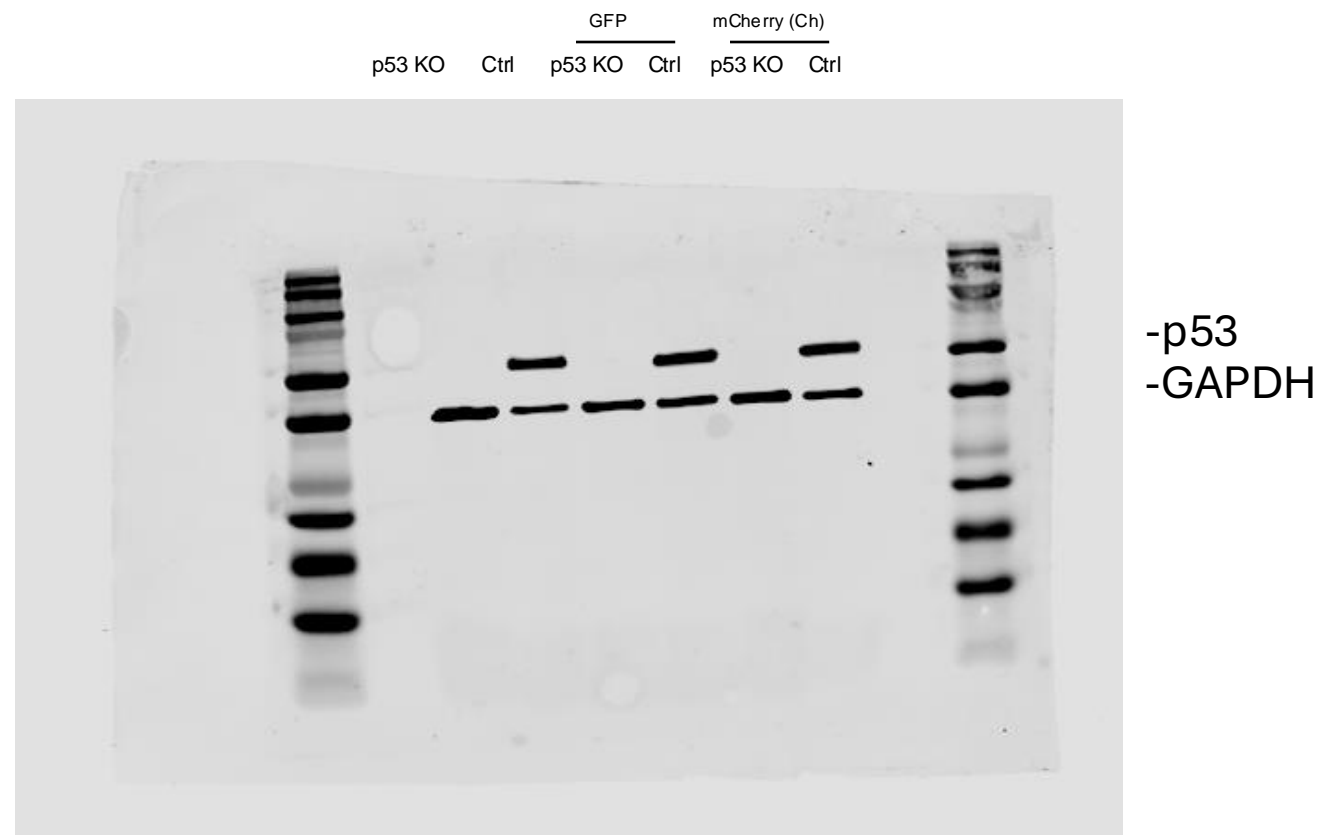

Supplementary Figure 1H

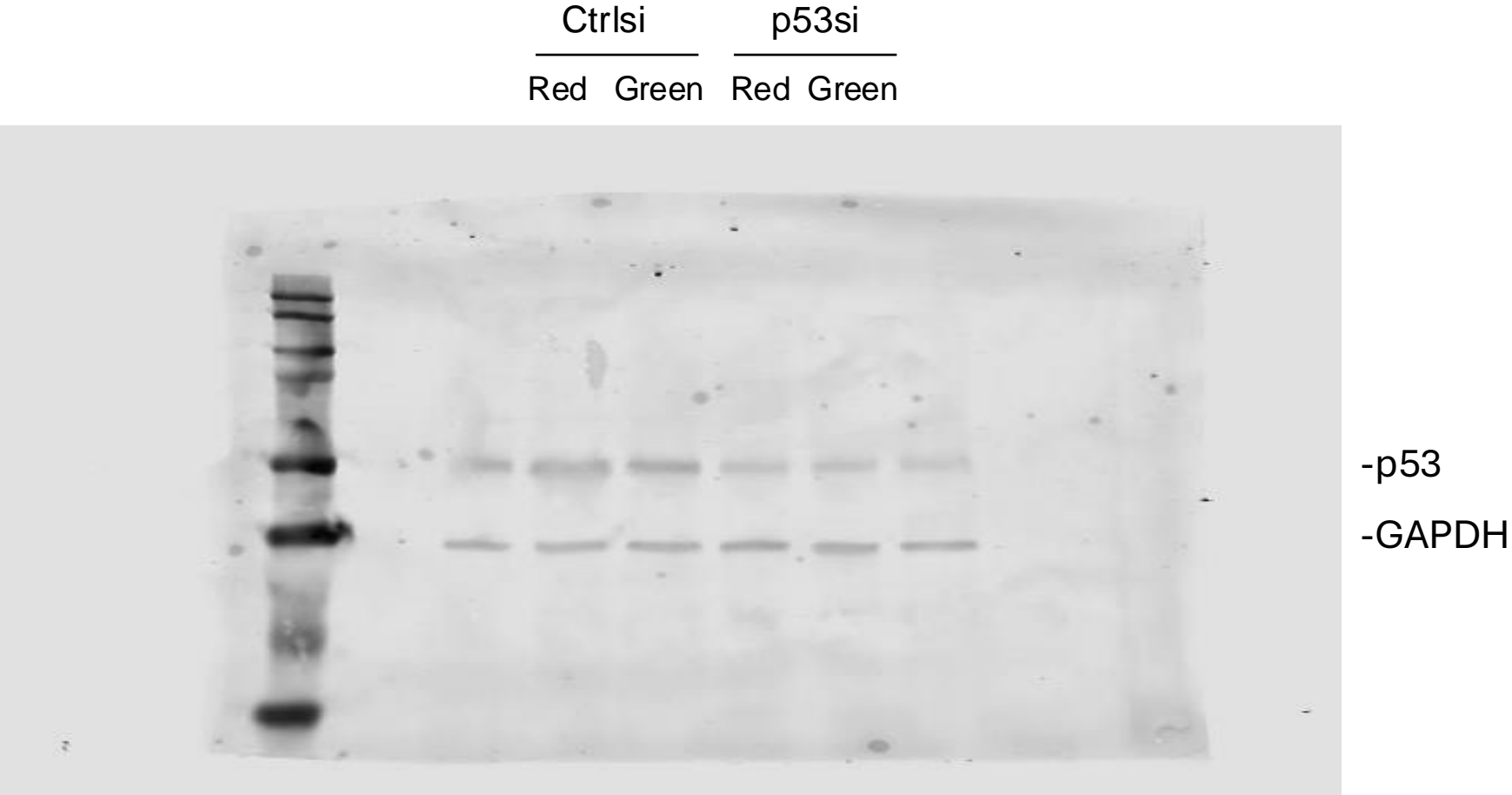

# Supplemental Figure 2A

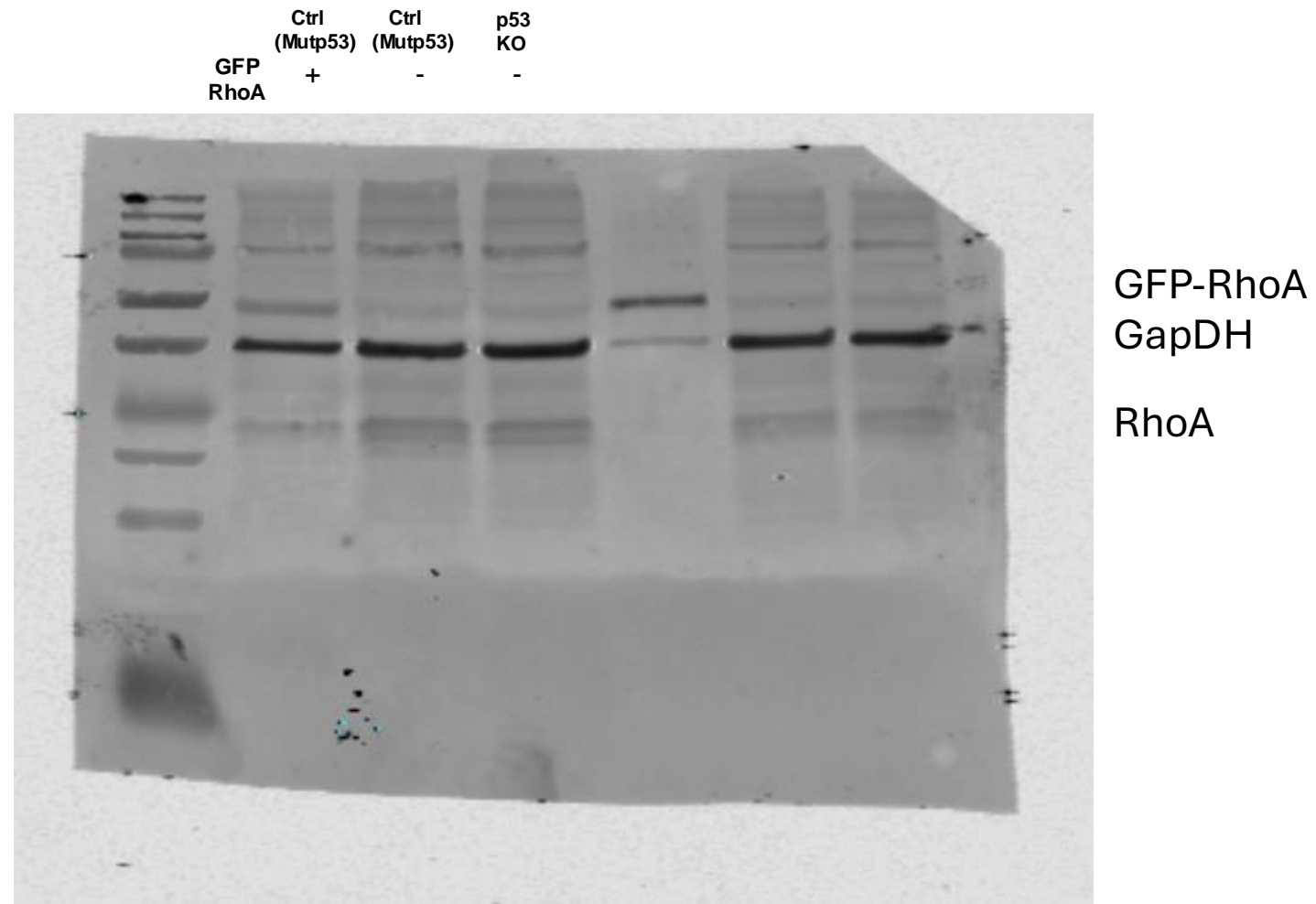

Supplement: Supplementary file 6 — original data [file 41420_2025_2582_MOESM6_ESM.pdf]
